# Local Visible‐Photocatalytic Production of Hydrogen and Warm Heat for Combination Treatment of Pressure Ulcer

**DOI:** 10.1002/advs.202503185

**Published:** 2025-06-10

**Authors:** Jiawei Zhu, Ting Chen, Wei Fang, Di Zhou, Dalong Ni, Bin Zhao, Yali Yang, Zhuobin Xu, Qianjun He

**Affiliations:** ^1^ Institute of Translational Medicine School of Medicine Yangzhou University Yangzhou 225001 P. R. China; ^2^ Guangdong Key Laboratory for Biomedical Measurements and Ultrasound Imaging National‐Regional Key Technology Engineering Laboratory for Medical Ultrasound School of Biomedical Engineering Shenzhen University Medical School Shenzhen 518060 P. R. China; ^3^ Department of Laser and Aesthetic Medicine Shanghai Ninth People's Hospital Shanghai Jiao Tong University School of Medicine Shanghai 200011 P. R. China; ^4^ Department of Radiology The First Affiliated Hospital of Chongqing Medical University Chongqing 401122 P. R. China; ^5^ Department of Orthopaedics Shanghai Key Laboratory for Prevention and Treatment of Bone and Joint Diseases Shanghai Institute of Traumatology and Orthopaedics Ruijin Hospital Shanghai Jiao Tong University School of Medicine Shanghai 200025 P. R. China; ^6^ Department of Dermatology Shanghai Ninth People's Hospital Shanghai Jiao Tong University School of Medicine Shanghai 200011 P. R. China; ^7^ Shanghai Key Laboratory of Hydrogen Science & Center of Hydrogen Science School of Materials Science and Engineering Shanghai Jiao Tong University Shanghai 200240 P. R. China; ^8^ Shenzhen Research Institute Shanghai Jiao Tong University Shenzhen 518057 P. R. China

**Keywords:** hydrogen therapy, nanocatalytic medicine, photocatalysis, pressure ulcer, wound healing

## Abstract

Pressure ulcer (PU) is hardly cured due to repeated pressures‐induced serious ischemia‐reperfusion injury (IRI), poor microcirculation, and chronical inflammation. Hydrogen molecule (H_2_) is proved as an emerging anti‐inflammatory and anti‐IRI agent with high biological safety, while local warm heating is well identified and able to improve tissue microcirculation for promoting wound repair. This work proposes a new strategy of visible‐photocatalytic hydrogen/warm heat production for combination treatment of PU, and develops palladium nanodots‐deposited hydrogen‐doped titanium dioxide nanorod (HTON‐Pd) as a novel multifunctional photocatalyst, achieving controllable and sustainable visible‐photocatalytic production of H_2_ and heat. Daily administration of the HTON‐Pd encapsulated hydrogel dressing (HTON‐Pd@Gel) with the assistance of visible light irradiation receives a high outcome of PU treatment. The stubborn PU wound has completely been cured after 11‐day treatment with HTON‐Pd@Gel. Mechanistically, hydrogen and warm heat photocatalytically produced by HTON‐Pd improve the PU wound microenvironment by synergistically promoting the proliferation and migration of skin cells, inhibiting their apoptosis, reducing the inflammatory response, and boosting the generation of new blood vessels in support of PU wound healing.

## Graphical abstract

A novel concept of visible‐photocatalytic hydrogen/warm heat production is proposed for combination treatment of pressure ulcer (PU), and develop a new kind of palladium nanodots‐deposited hydrogen‐doped titanium dioxide nanorods encapsulating hydorgel dressing to receive a high outcome of PU treatment by hydrogen/warm heat synergistically improving the PU wound microenvironment.

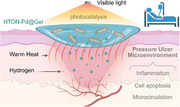

## 1 Introduction

Pressure ulcer (PU), called decubitus ulcer, is caused by persistent pressure on the skin, resulting in serious ischemia‐reperfusion injury (IRI), poor tissue microcirculation, and chronical inflammation.^[^ ^1^, ^2^, ^3^, ^4^ ^]^ With the development of aging population, the incidence rate of PU is rising, which has brought a serious burden to patients and the medical system. Currently, the clinical PU treatment methods mainly include drug therapy, surgical treatment, and physical therapy.^[^ ^1^, ^5^, ^6^, ^7^ ^]^ Although these three treatment methods have certain effects in relieving the pain and discomfort of patients with PU, they hardly cure PU completely because repetitive IRI, poor microcirculation, and chronical inflammation cannot be solved effectively. Therefore, there is an urgent need to develop new safe, and efficient treatment methods/drugs for PU.^[^ ^8^, ^9^, ^10^ ^]^

In recent years, hydrogen molecules (H_2_) have been proven to be a highly biosafe physiological regulator able to play antioxidative, anti‐inflammatory, and anti‐apoptotic roles in many inflammation‐related diseases.^[^ ^11^, ^12^, ^13^, ^14^, ^15^, ^16^ ^]^ Moreover, it has been found that H_2_ can reduce IRI by effectively scavenging cytotoxic reactive oxygen species (ROS) under catalysis of Fe‐porphyrin in the mitochondria, exhibiting a potential for PU treatment.^[^ ^17^, ^18^, ^19^, ^20^ ^]^ Furthermore, the mitigation of oxidative stress can support inflammation attenuation and promote the repair of injured tissues by some specific signaling pathways including Nrf2, AMPK, PI3K/Akt, etc.^[^ ^21^, ^22^, ^23^, ^24^, ^25^, ^26^, ^27^, ^28^ ^]^ Recently, we have verified that H_2_ gas inhalation can suppress the formation of PU by its anti‐IRI and anti‐inflammation functions to a certain extent.^[^ ^28^ ^]^ But the efficacy of PU therapy with H_2_ gas inhalation is not satisfied yet, because a long transport distance from the lung to the skin leads to considerably low H_2_ bioavailability of the PU skin. Therefore, local, sustainable, and effective delivery of H_2_ to the PU wound site is vitally important to high‐efficacy therapy of PU. The photocatalytic generation of H_2_ provides an ideal solution to PU therapy, which has not been reported so far.

Local warm heating of tissue by irradiation with an infrared lamp as a classical physical therapy method has been confirmed clinically effective to dilate blood vessels and promote tissue microcirculation by heat shock protein pathways in favor of wound repair.^[^ ^7^, ^29^, ^30^ ^]^ But the PU efficacy of heat therapy is still limited because the contribution of warm heating to both anti‐inflammation and anti‐IRI is very weak since warm heating cannot directly scavenge cytotoxic ROS. Therefore, the combination of hydrogen therapy with warm heating therapy might be a potential effective strategy for PU treatment, but has not been reported until now. It could be because it is difficult to combine these two therapeutic models effectively.

**Figure 1:**
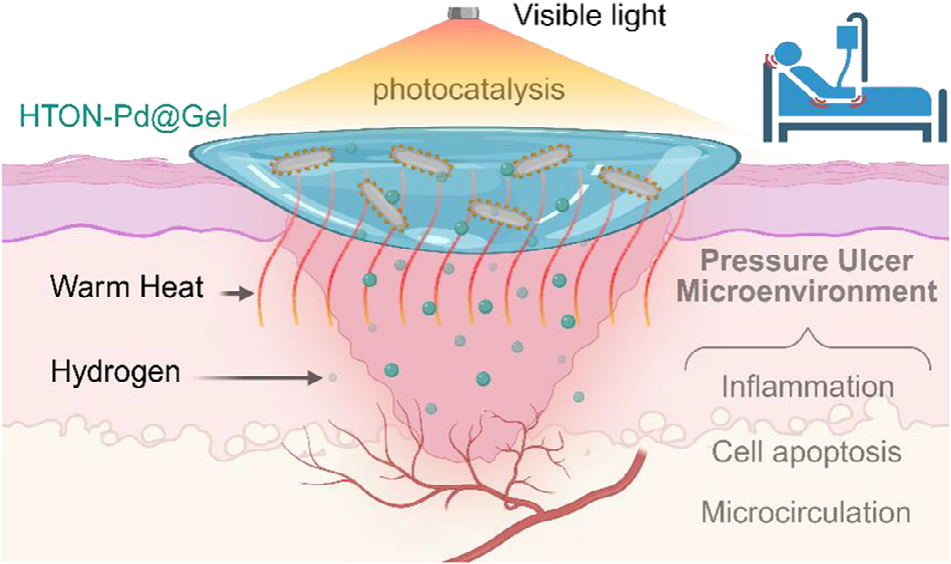
The schematical illustration of the proposed strategy of visible‐photocatalytic hydrogen/warm heat production for combination treatment of PU. Under daily irradiation of visible light on the HTON‐Pd@Gel covered PU wound, visible‐photocatalytically generated hydrogen and warm heat synergistically improved the PU wound microenvironment, including anti‐inflammation, anti‐apoptosis, pro‐proliferation, pro‐migration and pro‐angiogenesis for improved microcirculation, in support of accelerated PU wound healing.

In this work, we proposed an innovative strategy of combining photocatalytic hydrogen therapy with warm heating therapy for PU treatment, and developed palladium nanodots‐deposited hydrogen‐doped titanium dioxide nanorod (HTON‐Pd) as a new multifunctional therapeutic platform. The deposition of palladium nanodots on HTON can not only enhance the visible‐photocatalytic H_2_‐generating efficiency, but also enable visible‐photothermal therapy, achieving controllable and sustainable photo‐controlled production of H_2_ and heat. HTON‐Pd were encapsulated into a hydrogel to construct a novel dressing (HTON‐Pd@Gel), which exhibited an excellent outcome of PU therapy by hydrogen/warm heat synergistically improving the PU wound microenvironment (**Figure** **1**).

## 2 Results and Discussion

### 2.1 Synthesis and Structural Characterization of HTON‐Pd

Titanium dioxide (TON) is a typical kind of pharmaceutic adjuvant and suntan lotion additive with a high UV absorbancy, a UV‐photocatalytic ability, and a good biological safety. It is currently widely used as a white inorganic pigment in cosmetics. However, TON only absorbs short wavelength of UV light which has significant toxicity to cells and tissues. Long‐term exposure to UV will increase the risk of skin cancer. In this regard, this work attempted to transfer the absorption wavelength of TON from the UV range to the visible range, and doped hydrogen into TON to prepared a visible‐photocatalyst for H_2_ production by a full‐solution method. Rutile single‐crystal TON was first prepared by a hydrothermal method, and then HTON was synthesized by combining Li incorporation with Li/H exchange in an ethylenediamine solution at room temperature. Furthermore, in order to endow nanoparticles with a photothermal function and also enhance the electron/hole separation to improve the efficiency of visible‐photocatalytic hydrogen production,^[^ ^31^ ^]^ Pd nanodots were locally deposited on the surface of HTON nanorods at a high proportion (HTON‐Pd) by a UV‐photocatalyzed deposition method.^[^ ^32^, ^33^, ^34^ ^]^

**Figure 2:**
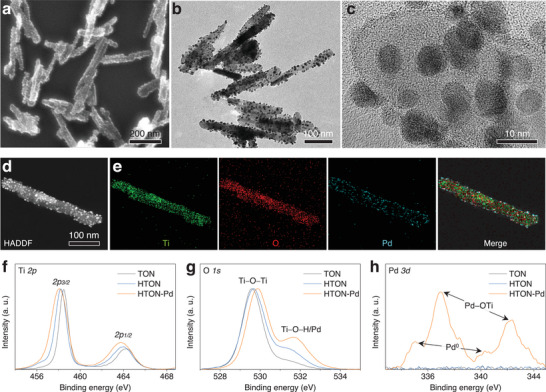
Morphology, composition and structure characterization of HTON‐Pd. SEM a), low‐magnification TEM b), and high‐resolution TEM c) images of HTON‐Pd, HADDF image d) and corresponding elemental mapping pattern e) of HTON‐Pd, and XPS patterns of HTON‐Pd f–h).

As shown in Figure S1 (Supporting Information), the as‐prepared TON exhibited a rod‐like morphology with a length of 80‒200 nm and a diameter of 26‒45 nm as well as a good dispersion in the aqueous solution. It can be seen from the X‐ray diffraction (XRD) data in Figure S2 (Supporting Information) and high‐resolution TEM images in Figure S3 (Supporting Information) that the synthesized TON had a single‐crystal structure of rutile (PDF#73‐1232). Such a single‐crystal rutile phase of TON will be favorable to stabilize hydrogen incorporation into TiO_2_ lattice.^[^ ^35^ ^]^ From high‐resolution TEM images in Figure S3 (Supporting Information), hydrogen incorporation caused the reduction of crystalline in accordance with XRD results in Figure S2 (Supporting Information). From SEM (**Figure** **2**a), TEM (Figure 2b,c), and HADDF (Figure S4, Supporting Information) images and elemental mapping patterns (Figure 2d,e), it is clearly visible that a large number of Pd nanodots with ≈5 nm in diameter were uniformly attached onto the surface of HTON‐Pd nanorods. EDX result in Figure S5 (Supporting Information) further indicated that the deposition amount of Pd onto HTON achieved 6.5% according to the molar Pd/Ti ratio of 0.049.

The X‐ray photoelectron spectroscopy (XPS) in Figure 2f and Figure S6 (Supporting Information) showed that compared with TON, the Ti *2p* peak of HTON shifted slightly toward lower energy, which was caused by the Ti─H bond, indicating the successful doping of hydrogen. In addition, at ≈531.6 eV, the O *1s* peaks of HTON‐Pd and HTON were much higher than that of TON (Figure 2g), indicating successful hydrogen incorporation and Pd deposition to form the bridging hydroxyl groups (Ti─OH─Ti) and the surface Ti─O─Pd binding. In addition to the formation of interfacial Ti─O─Pd binding, Pd mainly maintained Pd(0) nanodots (Figure 2h). The results indicated that Pd nanodots were stably deposited on the surface of HTON through interface binding owing to the virtue of local UV‐photocatalytic reduction. In addition, the dynamic light scattering (DLS) result revealed that as‐synthesized Pd‐HTON had a good dispersion in the aqueous solution (Figure S7, Supporting Information), which ensures that it can be evenly distributed in the hydrogel.

### 2.2 Visible‐Photocatalytic Hydrogen/Heat Production Performance

**Figure 3:**
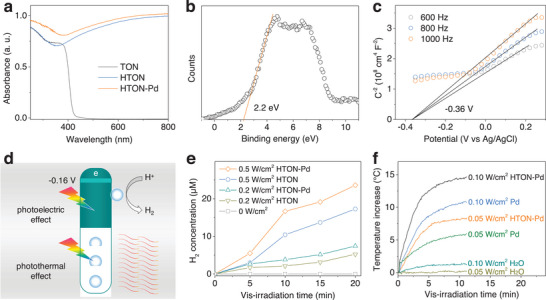
The electronic band structure characterization and visible‐photocatalytic hydrogen/heat generation performance measurement. UV–vis absorption spectra of TON, HTON and HTON‐Pd a), XPS valance band potential of HTON‐Pd b), Mott–Schottky plots of HTON‐Pd c), the schematic illustration of visible‐photocatalytic hydrogen/heat generation by HTON‐Pd d), visible‐photocatalytic hydrogen generation behavior of HTON‐Pd e), and visible‐photothermal performance of HTON‐Pd f).

The absorption spectra of TON, HTON, and HTON‐Pd in **Figure** **3**a showed that TON can only absorb UV light, while HTON exhibited strong absorbance in the VIS‐to‐NIR region due to hydrogen doping. Meanwhile, the absorbance of HTON‐Pd was generally stronger than that of HTON, owing to the local surface plasmon resonance (LSPR) effect of Pd nanodots (Figure 3a). Moreover, we examined the electronic band structure of HTON‐Pd to check whether HTON‐Pd can be competent for visible‐photocatalytic hydrogen production. According to the XPS valence band spectrum in Figure 3b, the valance band maximum (VBM) of HTON‐Pd relative to normal hydrogen electrode (NHE) was calculated to be 1.96 eV according to the following formula: VBM(versus NHE) = F+VBM(versus XPS)−4.44, where F is the work function of the instrument (4.20 eV). According to the Mott Schottky curve in Figure 3c, the conduction band minimum (CBM) value of HTON‐Pd relative to Ag/AgCl was measured to be −0.36 eV, and the CBM relative to NHE can be calculated to be −0.163 eV according to the formula: E(NHE) = E(Ag/AgCl)+0.197. Figure 3d illustrated the photocatalytic hydrogen/heat production mechanism of HTON‐Pd nanorods. The CBM potential of HTON‐Pd was lower than the reduction potential of H^+^/H_2_ (0 V) in support of visible‐photocatalytic hydrogen production. The VBM potential of HTON‐Pd was higher than the oxidation potential of GSH/GSSG (0.32 eV), and therefore we encapsulated GSH into HTON‐Pd@Gel as a hole sacrificial agent. Under the same illumination time and power of visible light, the photocatalytic hydrogen production efficiency of HTON‐Pd was distinctly higher than that of HTON (Figure 3e), which indicated that the surface deposition of Pd onto HTON can improve photocatalytic hydrogen production efficiency by enhancing the electron/hole separation. From Figure 3e, the Pd nanodot deposition increased the photocatalytic hydrogen production efficiency of HTON by 0.14 µm min^−1^ under 0.2 W cm^−2^ light irradiation, and 0.49 µm min^−1^ under 0.5 W cm^−2^ light irradiation. From the photothermal curves in Figure 3f, under the same irradiation conditions and at the same Pd concentration, the visible‐photothermal effect of HTON‐Pd was obviously stronger than that of Pd nanoparticles, possibly owing to the LSPR effect of Pd nanodots onto HTON. In addition, the visible‐photothermal effect was also dependent on the concentration of HTON‐Pd (Figure S8, Supporting Information). Moreover, during repeating visible light irradiation, the visible‐photocatalytic generation of hydrogen and heat were stable in spite of different power densities of visible light as the increases of H_2_ and heat generation were linearly correlated to visible‐irradiation time (Figure S9, Supporting Information). By linear fitting, the efficiency of hydrogen generation was calculated to be 0.31 µm min^−1^ under 0.5 W cm^−2^ light irradiation, while the efficiency of heat generation was calculated to be 1.04 °C min^−1^ in spite of light density (Figure S9, Supporting Information).

### 2.3 In Vitro Photocatalytic Hydrogen/Warm Heat Combined Therapy Performances of HTON‐Pd

On the basis of verifying that HTON‐Pd can achieve visible‐photocatalytic production of hydrogen and heat, the effect of hydrogen and warm heat combined therapy was further explored at the cellular level. In the concentration range of 0–200 µg mL^−1^, Pd‐HTON did not show obvious cytotoxicity to human skin fibroblasts (HSF) and human immortalized keratin‐forming cells (HaCaT), meaning that HTON‐Pd had high cytocompatibility (Figure S10, Supporting Information). In order to eliminate the interference of GSH on cell experiments, GSH with different concentrations was co‐incubated with HSF and HaCaT cells. As shown in Figure S11 (Supporting Information), GSH did not affect the viability of HSF and HaCaT cells in the concentration range of 0–250 µg mL^−1^.

**Figure 4:**
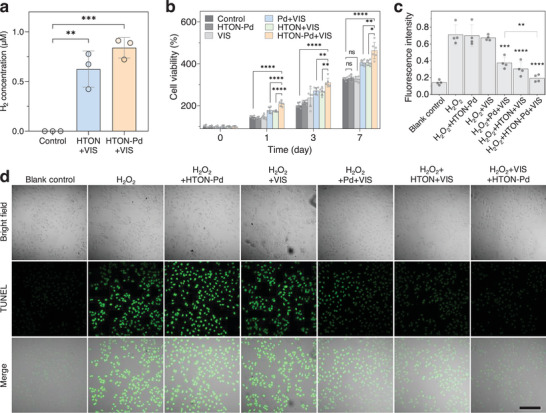
In vitro effect of visible‐photocatalytic hydrogen/warm heat production on the proliferation and oxidative stress‐induced apoptosis of skin cells. Intracellular visible‐photocatalytic H_2_ generation (*n*=3 biologically independent samples) a), the effect of hydrogen and warm heat treatments on the proliferation of HSF cells (*n* = 6 biologically independent samples) b), the effect of hydrogen and warm heat treatments on the oxidative stress‐induced apoptosis of HaCaT cells (scale bar, 200 µm) d), and corresponding statistical analysis (*n* = 4 biologically independent samples) c). The value of *p* < 0.05 was considered to be a significant difference (^*^ *p* < 0.05, ^**^ *p* < 0.01, ^***^ *p* < 0.001, and ^****^ *p* < 0.0001; ns, no significant difference).

**Figure 5:**
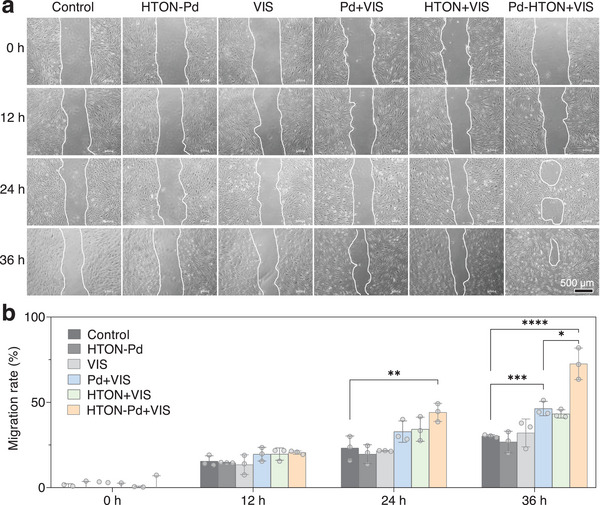
In vitro effect of visible‐photocatalytic hydrogen and warm heat production on the migration of skin cells. Digital images of HSF cells simulated wounds after various treatments for different time periods a), and corresponding statistical analysis (*n*=3) b). The value of *p* < 0.05 was considered to be a significant difference (^*^ *p* < 0.05, ^**^ *p* < 0.01, ^***^ *p* < 0.001, and ^****^ *p* < 0.0001; ns, no significant difference).

Furthermore, we examined visible‐photocatalytic hydrogen production of HTON‐Pd in cells. From **Figure** **4**a, it can be seen that visible light irradiation (0.05 W cm^−2^, 15 min) on HTON‐Pd (200 µg mL^−1^) treated HSF cells can effectively produce H_2_ in cells. It can be seen from Figure 4b and Figure S12 (Supporting Information) that individual hydrogen (HTON+VIS) and warm heat (Pd+VIS) treatments can promote the proliferation of both HSF and HaCaT cells, but neither HTON‐Pd nor VIS treatment can. After 30 min of H_2_O_2_ treatment, HaCaT cells apoptosis was obvious, owing to oxidative dagame to cells. Hydrogen and warm heat can inhibit the apoptosis of HaCaT cells (Figure 4c,d). At the same time, both hydrogen and warm heat can also boost the migration of HSF and HaCaT cells (**Figure** **5**; Figure S13, Supporting Information). By comparison to single treatment, hydrogen/heat combined treatment can achieve higher pro‐proliferation, pro‐migration, and anti‐apoptosis effects. These results suggested that the HTON‐Pd medicated visible‐photocatalytic generation of hydrogen and warm heat was in great favor of repairing oxidatively damaged skin wounds such as PU.

### 2.4 In Vivo Photocatalytic Hydrogen/Warm Heat Therapy Outcomes

Based on the above in vitro results, we further evaluated the effect of hydrogen/warm heat combined therapy on wound healing in a PU wound model. For treatment of PU, HTON‐Pd nanorods were mixed in a chitosan/hydrochloric acid hydrogel to prepare a mixed dressing (HTON‐Pd@Gel) which can be adhered to the surface of PU wound. In addition, 15 min visible light irradiation (0.05 W cm^−2^) did not change the microstructure (Figure S14, Supporting Information) and rheological behavior (Figure S15, Supporting Information) of the HTON‐Pd@Gel hydrogel obviously, which should be in favor of stable photocatalytic treatment.

**Figure 6:**
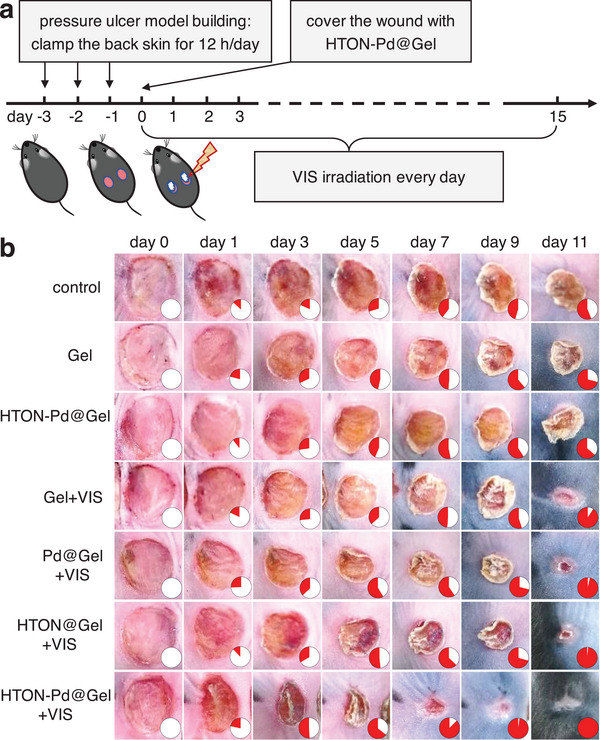
In vivo performance of hydrogen/warm heat combined treatment of PU. A schematic illustration of the PU model building and the treatment procedure with HTON‐Pd@Gel a), and representative digital images of PU wounds during treatments b). The red pie chart in figure b corresponds to the wound healing percentage.

As illustrated in **Figure** **6**a, the PU model was built by clamping the back skin of mice using two circular magnetic plates for 12 h followed by release for 12 h. This clamping/release circulation was repeated three times, and finally, two circular wounds were obtained on the back of mice. After modeling, the PU wound surface was coated with the HTON‐Pd@Gel dressing and then locally irradiated with a Xenon lamp (0.05 W cm^−2^, 15 min per time, three times per day). According to Figure S16 (Supporting Information), after illumination for 15 min, the temperature on the PU wound in the HTON‐Pd@Gel group mice increased by ≈5 °C, indicating that HTON‐Pd@Gel has a good visible‐responsive warm heating performance which meets the warm heating requirement. Furthermore, after a 24‐h exposure to visible light, both adsorption (Figure S17, Supporting Information) and crystal phase (Figure S18, Supporting Information) of HTON‐Pd@Gel kept unchanged, indicating high stability of HTON‐Pd@Gel without the light‐induced degradation and leaching of HTON and Pd. It is also worth noting that different from general NIR‐photothermal therapy which utilizes blood vessels in the depth of tissue as a photothermal emission source,^[^ ^36^ ^]^ visible light used in this work does not need to penetrate across the hydrogel dressing or even into the depth of tissue for heat generation. At the used encapsulation proportion of 1 mg mL^−1^ in this work, visible light can easily penetrate across the HTON‐Pd@Gel dressing as shown in Figure S17a (Supporting Information), ensuring the HTON‐Pd‐medicated photocatalytic generation of hydrogen and heat. Moreover, high portable visible light sources can be used in this therapeutic system, which will enhance its translational relevance with high patient compliance.

From Figure 6b and Figure S19 (Supporting Information), it can be seen that on the 7^th^ day of treatment with HTON‐Pd@Gel+VIS, the wound scab shed off with a wound healing rate of up to 79%, while the scabs in the other treatment groups of mice did not. On the 9^th^ day of treatment, the healing rates of PU wound in the control, Pd@Gel+VIS, HTON@Gel+VIS, and HTON‐Pd@Gel+VIS groups achieved to 55%, 74%, 73%, and 96%, respectively. It indicated that both warm heat and hydrogen molecules can promote the repair of PU, but hydrogen/heat combined therapy with HTON‐Pd@Gel+VIS had the highest efficiency in promoting PU wound healing, exhibiting a combined therapeutic effect of hydrogen and warm heat. It was clearly visible that after treatment with HTON‐Pd@Gel+VIS for 11 days, PU wounds were almost completely healed, while the control group without use of any dressing was far away from healing (Figure 6b).

### 2.5 Photocatalytic Generation of Hydrogen/Warm Heat Improves PU Wound Microenvironment

**Figure 7:**
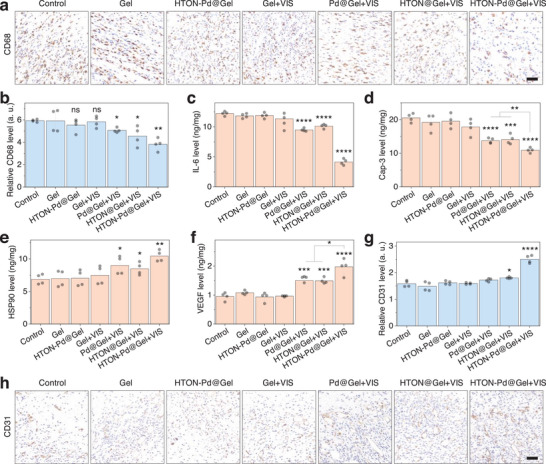
The regulatory effect of hydrogen and warm heat on factors expression. Immunohistochemical staining of CD68 expression after 6‐day treatment a) and corresponding statistical results (*n* = 3 biologically independent samples) (scale bar, 50 µm) b), ELISA analysis of IL‐6 c), Caspase‐3 d), HSP90 e) and VEGF f) expressions at the PU wound site after 6‐day treatment (*n* = 4 biologically independent samples), immunohistochemical staining of CD31 expression after 6‐day treatment g), and corresponding statistical analysis (*n* = 3 biologically independent samples) (scale bar, 50 µm) h). The value of *p* < 0.05 was considered to be a significant difference (^*^ *p* < 0.05, ^**^ *p* < 0.01, ^***^ *p* < 0.001, and ^****^ *p* < 0.0001; ns, no significant difference).

Persistent pressure on the skin causes serious oxidative stress and damage, chronical inflammation and poor tissue microcirculation lack of angiogenesis, which together form a sever microenvironment of PU to prevent from skin self‐healing. Based on the above excellent therapeutic outcome, we further investigated the effect of photocatalytic hydrogen/heat combined therapy on the PU microenvironment. CD68 is a biomarker of macrophage pro‐inflammatory activity. Immunohistochemistry was used to analyze the expression of CD68 at the site of PU wound. From **Figures** **7**a,b and S20 (Supporting Information), both individual warm heat (Pd@Gel+VIS group) and individual hydrogen molecule (HTON@Gel+VIS) can slightly decrease the expression of CD68 at the PU wound site, but the combined hydrogen/warm heat treatment with HTON‐Pd@Gel+VIS can more effectively and sustainedly block inflammation by reducing the expression of CD68. From Figure 7c, it can be seen that the combined hydrogen/warm heat treatment also more obviously inhibit the expression of IL‐6 at the wound site of PU compared with individual hydrogen and heat treatments in accordance with the results about CD68. Moreover, such an anti‐inflammation effect of combined therapy was stronger at the early stage of PU (Figure 7c; Figure S21, Supporting Information), possibly owing to higher inflammation phenotype in the initial. Caspase‐3 is an executing molecule in the process of cell apoptosis, and different apoptotic pathways can activate Caspase‐3 and cause cell apoptosis. From Figure 7d, neither Gel nor HTON‐Pd@Gel can significantly reduce the expression of Caspase‐3 at the wound site of PU, but the combined effect of hydrogen and warm heat made bigger contribution to Caspase‐3 inhibition than individual hydrogen and heat treatments, indicating that the hydrogen/warm heat combined treatment can inhibit the apoptosis of cells at the PU wound. Similarly, the expression of HSP90 protein in the HTON‐Pd@Gel+VIS group was more significantly enhanced (Figure 7e), which promoted the protection of cells from apoptosis. Angiogenesis is essential in wound healing and tissue regeneration, and CD31 is a biomarker for endothelial cell differentiation and VEGF is a classic vascular endothelial growth factor. Therefore, we performed the immunohistochemical staining of CD31 and measured the level of VEGF at the wound site of PU after treatment for 6 days. From Figure 7f, it can be found that both hydrogen and warm heat treatments can induce the expression of VEGF but Gel to a certain extent, HTON‐Pd@Gel and Gel+VIS did not, while the hydrogen/warm heat combined effect was more significant. Such a combination effect seemed more distinct to promote the expression of CD31 (Figure 7g,h).

As a result, the HTON‐Pd@Gel+VIS treatment more in‐time and effectively reformed the fibrosis during PU wound healing and induced the regeneration of skin (Figures S22 and S23, Supporting Information). These results coordinately indicated that the hydrogen/warm heat combined treatment with HTON‐Pd@Gel+VIS can more effectively block the tissue inflammation, inhibit the cell apoptosis, and promote vascular regeneration in support of accelerated wound healing.

In addition, in order to verify the biological safety of hydrogen/warm heat combined therapy with HTON‐Pd@Gel+VIS in vivo, the body weight of mice was monitored during the whole treatment process, and blood samples were collected for biochemical testing at the end of treatment. Figure S24 (Supporting Information) showed that there was no significant difference in body weight among all the treated groups. From Figures S25 and S26 (Supporting Information), it can be seen that the representative indicators of all the blood samples remained within the normal range, indicating that the hydrogen/warm heat combined treatment exhibited a high biological safety. Furthermore, mice were euthanized after treatment, and their heart, liver, spleen, lung, and kidney were collected for H&E staining. From Figure S27 (Supporting Information), it can be observed that all the experimental groups did not cause significant damage to these main organs. Meanwhile, neo‐tissues at the PU wound site were collected after HTON‐Pd@Gel+VIS treatment for two weeks to evaluate the levels of ROS and various inflammatory cytokines. From Figure S28 (Supporting Information), HTON‐Pd@Gel+VIS treatment did not significantly affect the levels of ROS and various inflammatory cytokines after treatment for two weeks compared with the case of normal mice without PU wound, suggesting that HTON‐Pd@Gel had high biosafety after complete PU wound healing. In addition, the result of ICP measurement indicated that HTON‐Pd was mainly distributed in the scab rather than the blood and major organs (Figure S29, Supporting Information), indicating that HTON‐Pd was possibly included the scab during wound healing and excreted from the body along with the shedding of scab. These results further confirmed the high biological safety of HTON‐Pd@Gel+VIS treatment.

## 3 Conclusion

In conclusion, in view of the pathological characteristics of the PU wound, we developed the HTON‐Pd nanorods encapsulated hydrogel as a visible‐photocatalyst, which can generate hydrogen and warm heat in the PU wound under irradiation of visible light. Under the combined action of hydrogen and warm heat, the inflammation was well controlled at the PU wound site and the generation of blood vessels was promoted in favor of tissue microcirculation improvement. This work proposes a combined treatment strategy of hydrogen and warm heat to solve many issues in the current treatment of pressure ulcers and provides a simple, safe, and efficient solution for the treatment of pressure ulcer.

## 4 Experimental Section

### Synthesis of TON

First, 20 mL of deionized water was placed in an ice bath. Under stirring at a speed of 500 r min^−1^, 2.2 mL of titanium tetrachloride (TiCl_4_) was added dropwise to the aforementioned ice water to form a titanium tetrachloride aqueous solution. Subsequently, the mixture was continuously stirred at a speed of 500 r min^−1^ for 30 min and then placed in a 50 mL high‐pressure autoclave. The high‐pressure autoclave was placed in an oven and heated to 220 °C at a rate of 10 °C min^−1^ followed by thermal retardation for another 2 h. After that, the autoclave was cooled to room temperature, and a white precipitate was collected by centrifugation, washed three times with deionized water and anhydrous ethylenediamine in order, and finally dispersed in anhydrous ethylenediamine.

### Synthesis of HTON

Under argon atmosphere protection and stirring, 140 mg of metal lithium foil was completely dissolved in 15 mL of anhydrous ethylenediamine to form an electronic solution. The above‐prepared ethylenediamine solution of TON (5 mL, 40 mg mL^−1^) was added to the electronic solution, and then stirred continuously for 6 days. After sufficient reaction, 50 mL of hydrochloric acid (1 mol L^−1^) was added slowly to the reaction solution to quench excessive electrons. A black precipitate was collected by centrifugation, washed with deionized water three times, and dispersed in water.

### Synthesis of HTON‐Pd

First, 1.8 mg of sodium tetrachloropalladium (Na_2_PdCl_4_) was dissolved in 10 mL of deionized water. Methanol (8 mL) and the aqueous solution of HTON (15 mL, 10 mg HTON) were added in a 100 mL glass bottle, and mixed evenly under sonication for 10 min in an ultrasonic cleaner. The above two solutions were mixed quickly, and sonicated for 10 min in the ultrasonic cleaner. Then, the air in the bottle was replaced with nitrogen gas. Next, the mixed solution was illuminated with ultraviolet light for 5.5 h under magnetic stirring at a speed of 500 r min^−1^. Finally, the product HTON‐Pd was collected by centrifugation and washed with deionized water three times followed by dispersion in water.

### Synthesis of HTON‐Pd@Gel

In order to construct the HTON‐Pd@Gel hydrogel dressing, 2% chitosan (CS) was prepared in 0.1 m hydrochloric acid, 10% β‐glycerol phosphate (GP) was prepared in deionized water, and 1% sodium hyaluronate (HA) was prepared in deionized water. Subsequently, three solutions were mixed at a volume ratio of 5:3:2, and the aqueous solution of HTON‐Pd was added under the assistance of shaking to achieve a concentration of 1 mg mL^−1^ HTON‐Pd. Then, the mixed hydrogel was placed in a 37 °C water bath for thermal retardation for 10 min. Regarding the treatment of PU model, newly prepared HTON‐Pd@Gel was immediately covered on the PU wound so that it can be naturally gelated on the wound through body temperature.

### Characterization of HTON‐Pd

The morphology and size of HTON‐Pd were characterized using a high‐resolution scanning electron microscopy (Thermo APREO S) and a tungsten‐filament transmission electron microscopy (HITACH HT7700), and the elemental distribution of HTON‐Pd was detected using a field‐emission transmission electron microscopy (JEM‐F200). The crystal structure of HTON‐Pd was detected using a powder X‐ray diffractometer (XRD, M21X, room temperature, 40 kV, 200 mA) within a scanning range of 20–90°. The UV absorption spectrum of HTON‐Pd was recorded using a UV spectrophotometer. The valence band spectrum of HTON‐Pd was detected using an X‐ray photoelectron spectroscopy analyzer (PerkinElmer PHI 5000C). The Mott Schottky curves of HTON‐Pd at different frequencies were measured on an electrochemical workstation (CHI 760E). The particle size distribution was measured by DLS on a Malvern Mastersizer 3000.

### Light Stability Assessment of HTON‐Pd@Gel

The as‐synthesized HTON‐Pd@Gel was drop onto a clean glass to from a uniform film. Then, the film was irradiated with a Xenon lamp (0.05 W cm^−2^) with a fixed time duration (0, 12, and 24 h). Finally, the absorption spectra and XRD patterns of the film were collected to evaluate the light stability of HTON‐Pd@Gel.

A rotational rheometer (ARES‐G2, TA Instruments) was employed to measure the storage (elastic) modulus (G′) and loss (viscous) modulus (G′′) at 37 °C to investigate the rheological behavior of the HTON‐Pd@Gel hydrogel before and after 15 min visible light irradiation (0.05 W cm^−2^). In brief, the disc‐shaped sample with 0.5 mm in thickness and 8 mm in diameter was first adhered to the metal plate and substrate with a super glue in a circular metal tank which can control the temperature of bath solution. Then, the sample was surrounded by water to prevent water evaporation from the sample during measurement. A rheological frequency sweep test was performed from 0.1 to 300 rad s^−1^ with a shear strain of 0.1% in the parallel‐plates geometry.

### Visible Light‐Catalytical Hydrogen Production Measurement

First, GSH (1.5 mL, 20 mmol L^−1^) was dissolved into an aqueous solution of HTON or HTON‐Pd (1.5 mL, 4 mg mL^−1^). The air in the bottle was replaced with argon gas for 10 min, and the bottle with the mixed solution was sealed. The above‐mixed solution was irradiated with a xenon lamp (CHF‐XM500, 400–800 nm), and the generation of hydrogen gas was detected using a gas chromatograph at fixed time points (0, 5, 10, 15, and 20 min).

### In Vitro Photothermal Experiment

The aqueous solutions of HTON‐Pd and Pd at the same Pd concentration were irradiated under the xenon lamp (CHF‐XM500, 400–800 nm) at different power densities (0.05 and 0.01 W cm^−2^), and the temperature change of sample solutions was recorded in real‐time. The photothermal curves of HTON‐Pd and Pd were plotted.

### Measurement of Cytotoxicity

Human skin fibroblast (HSF) and human immortalized keratin‐forming (HaCaT) cell lines were purchased from Procell Life Science & Technology Co., Ltd. (Wuhan, China). HSF and HaCaT cells were cultured in the DMEM medium (Gibco, USA) containing 10% fetal bovine serum (FBS, Gibco, USA). To study the cytotoxicity of HTON‐Pd on two types of cells, cells (1 × 10^4^ cells well^−1^) were seeded in 96‐well plates and incubated with the medium containing different concentrations of HTON‐Pd (0, 25, 50, 100, and 200 µg mL^−1^) for 24 h. 10 µL CCK‐8 reagent (Beyotime, Shanghai, China) was added to each well, incubated at 37 °C for 60 min, and then the absorbance at 450 nm was collected on a BioTek Synergy HIM microplate reader (CA, USA).

### Determination of the Effect of GSH on Cell Survival Rate

To test the effect of GSH on cell survival rate, cells (1 × 10^4^ cells well^−1^) were seeded in 96‐well plates and incubated with the medium containing different concentrations of GSH (0, 62.5, 125, and 250 µg mL^−1^) for 24 h. 10 µL CCK‐8 reagent (Beyotime, Shanghai, China) was added to each well, incubated at 37 °C for 60 min, and then the absorbance at 450 nm was collected on the BioTek Synergy HIM microplate reader (CA, USA).

### Intracellular Hydrogen Release Measurement

First, 200 µg mL^−1^ HTON‐Pd was incubated with HSF cells for 24 h. Cells were digested gently, collected by centrifugation, and resuspended in PBS. The cell solution was put in a bottle, which was then sealed and irradiated with the xenon lamp for 15 min. Finally, GC (GC‐2030, Shimadzu) was used to detect the amount of hydrogen generated in cells.

### Measurement of Cell Proliferation

To detect cell proliferation, cells (1 × 10^3^ cells well^−1^) were seeded in 96‐well plates and incubated with the medium containing of 200 µg mL^−1^ HTON‐Pd for 7 days. For VIS‐involving groups, cells were irradiated with the xenon lamp at a power of 0.05 W cm^−2^ for 5 min per day, which was repeated for 3 times per day. The CCK‐8 method was used to detect cell viability at different time points (0, 1, 3, and 7 days).

### Measurement of Cell Migration

To detect cell migration, cells were seeded in 6‐well plates, and then a sterile 10 µL pipette tip was used to create a wound scratch in the middle of the orifice plate to simulate before cells were overgrown. Cells were cultured in the medium containing 200 µg mL^−1^ HTON‐Pd, and irradiated with the xenon lamp at a power of 0.05 W cm^−2^ for 5 min per day, which was repeated for 3 times per day. After incubation for 12, 24, and 36 h, cell migration was observed under an inverted microscope (Nikon, FHEIPSE, Japan). ImageJ software was used to calculate the relative migration area compared to the blank control without HTON‐Pd treatment.

### Measurement of Cell Apoptosis

To detect cell apoptosis, cells were seeded in a confocal dish. After cell adhesion, each dish was treated with 100 µL hydrogen peroxide at a final concentration of 100 µmol L^−1^ for 30 min. After that, the dish was rinsed for 3 times with PBS immediately. Culture medium was replaced with one containing HTON‐Pd, and then irradiated with the xenon lamp for 5 min, which was repeated for 3 times a day. Finally, the one‐step TUNEL apoptosis assay kit (Beyotime) was used to detect the impact of hydrogen/hyperthermia on cell apoptosis and to observe cell apoptosis under an ultrahigh resolution laser confocal microscope (Germany, CARL ZEISS).

### Establishment of PU Animal Model

All the animal experiments followed the protocol approved by the Animal Care and Use Committee of Shenzhen University (No. 2021005). Female C57BL/6J mice (8 weeks old) were purchased from Sibeifu Beijing Biotechnology Co., Ltd. Mice were raised in an environment with a light/dark cycle at 22–25 °C for 12 h and provided with normal food and drinking water. After one week, the back of mice was depilated, and disinfected with an alcohol cotton pad. The back skin (including epidermis, dermis, subcutaneous fat, and some loose connective tissue, without muscle) was gently lifted, clamped with two circular magnetic discs (with a diameter of 12 mm and a thickness of 1.5 mm) for 12 h, and then released for 12 h. This clamping‐releasing operation was repeated for three days, and finally, two circular PU wounds were created on the back of mice.

### In Vivo Photothermal Experiment

Fresh composite sol (HTON‐Pd@Gel) was coated onto the PU wound. After ≈15 min, the PU wound was irradiated under the xenon lamp at a power density of 0.05 W cm^−2^. Meanwhile, the temperature at the site of PU wound was recorded in real‐time using a thermal imager (FLIR A300‐series). The curve of temperature change at the PU wound site was plotted.

### Treatment of PU Animal Model

Model mice were randomly divided into seven groups (*n* = 6 per group, biologically independent samples) for various treatments, 1) control group, 2) Gel group, 3) HTON‐Pd@Gel group, 4) Gel+VIS group, 5) Pd@Gel+VIS group, 6) HTON @Gel group, and 7) HTON‐Pd@Gel+VIS group. For the HTON‐Pd@Gel+VIS group, fresh composite sol (HTON‐Pd@Gel) was covered onto the PU wound of mice, which was then irradiated with the xenon lamp every day (15 min each time, repeated 3 times, 0.05 W cm^−2^). The digital photos of wound were collected at fixed time points and ImageJ software was used to calculate the size of wound. After HTON‐Pd@Gel+VIS treatment for two weeks, the neo‐tissues at the PU wound site were collected to evaluate the levels of ROS and various inflammatory cytokines (IL‐6, IL‐1β, TNF‐α, IL‐10, and TGF‐β) by related assay kits. Meanwhile, the normal skin tissue in the normal mice was collected for comparison. In addition, main organs, blood, and scab were collected for ICP measurement to evaluate the biodistribution of Pd.

### Evaluation of Liver/Kidney Function and Systematic Toxicity

On the last day of treatment, blood was collected for analyzing liver and kidney function indicators and blood routine. In addition, main organs of mice were extracted for H&E staining and imaging.

### Statistical Analysis

All experimental data were expressed as mean ± SD. Statistical analyses were performed with GraphPad Prism 8 and Origin 2021. The two‐tailed Student's *t* test method was used for the comparison between two groups. The value of *p* < 0.05 was considered to be a significant difference (^*^ *p* < 0.05, ^**^ *p* < 0.01, ^***^ *p* < 0.001, and ^****^ *p* < 0.0001; ns, no significant difference). The schematical illustration in Figure 1 was drawn with BioRender.com.

## Conflict of Interest

The authors declare no conflict of interest.

## Supporting information



Supporting Information

## Data Availability

The data that support the findings of this study are available from the corresponding author upon reasonable request.

## References

[1] Z. S. Razavi , S. A. Sharafshadehi , M. H. Yousefi , F. Javaheri , M. R. R. Barghani , H. Afkhami , F. Heidari , Arch. Dermatol. Res. 2025, 317, 320. 39888392 10.1007/s00403-024-03790-8

[2] K. K. Zajac , K. Schubauer , R. Simman , J. Wound Care 2024, 33, S18. 10.12968/jowc.2024.0079 39283887

[3] C. E. Fife , E. Gkotsoulias , Adv. Wound Care 2019, 8, 580. 10.1089/wound.2018.0905 PMC6906755 31832271

[4] L. Jiang , Q. Tu , Y. Wang , E. Zhang , Ostomy Wound Manag. 2011, 57, 55. 21350273

[5] D. Duscher , E. Neofytou , V. W. Wong , Z. N. Maan , G. C. Gurtner , Proc. Natl. Acad. Sci. USA 2015, 112, 94. 25535360 10.1073/pnas.1413445112 PMC4291638

[6] R. Houwing , M. Overgoor , M. Kon , G. Jansen , B. S. van Asbeck , J. R. Haalboom , J. Wound Care 2000, 9, 36. 10827667 10.12968/jowc.2000.9.1.25939

[7] Haesler E. , W. Healing , M. Collaborative , Wound Pract. Res. 2023, 31, 90.

[8] G. Xie , X. Wang , M. Mo , L. Zhang , J. Zhu , Macromol. Biosci. 2023, 23, 2200378. 10.1002/mabi.202200378 36337010

[9] P. Liu , Y. Fu , F. Wei , T. Ma , J. Ren , Z. Xie , S. Wang , J. Zhu , L. Zhang , J. Tao , J. Zhu , Adv. Sci. 2022, 9, 2202591. 10.1002/advs.202202591 PMC9443460 35839467

[10] S. Du , N. Zhou , G. Xie , Y. Chen , H. Suo , J. Xu , J. Tao , L. Zhang , J. Zhu , Nano Energy 2021, 85, 106004.

[11] Z. Jin , P. Zhao , W. Gong , W. Ding , Q. He , Nano Res. 2023, 16, 2020.

[12] I. Ohsawa , M. Ishikawa , K. Takahashi , M. Watanabe , K. Nishimaki , K. Yamagata , K.‐i. Katsura , Y. Katayama , S. Asoh , S. Ohta , Nat. Med. 2007, 13, 688. 17486089 10.1038/nm1577

[13] B. Zhao , L. Zeng , D. Chen , S. Xie , Z. Jin , G. Li , W. Tang , Q. He , Sci. Adv. 2022, 8, abq0959. 10.1126/sciadv.abq0959 PMC9534508 36197972

[14] A. Wu , L. Jiang , C. Xia , Q. Xu , B. Zhou , Z. Jin , Q. He , J. Guo , Adv. Sci. 2023, 10, 202303016. 10.1002/advs.202303016 PMC10558630 37587791

[15] H. Liu , D. Chen , X. Yang , M. Zhao , J. Zhong , W. Ding , W. Hu , H. Yang , Z. Wang , Q. He , Adv. Funct. Mater. 2024, 34, 2316227.

[16] M. Zhao , Z. Jin , C. Xia , S. Chen , L. Zeng , S. Qin , Q. He , Biomaterials 2023, 301, 122230. 37418855 10.1016/j.biomaterials.2023.122230

[17] S. Chen , Y. Zhu , Q. Xu , Q. Jiang , D. Chen , T. Chen , X. Xu , Z. Jin , Q. He , Nat. Commun. 2022, 13, 5684. 36167814 10.1038/s41467-022-33475-7 PMC9515190

[18] Q. Xu , S. Chen , L. Jiang , C. Xia , L. Zeng , X. Cai , Z. Jin , S. Qin , W. Ding , Q. He , Natl. Sci. Rev. 2023, 10, nwad063. 37056424 10.1093/nsr/nwad063 PMC10089581

[19] Y. Zhu , Q. Jiang , Z. Jin , D. Chen , Q. Xu , J. Chen , Y. Zeng , S. Chen , Q. He , Adv. Healthcare Mater. 2023, 12, 2201705. 10.1002/adhm.202201705 36546774

[20] S. Chen , Y. Yu , S. Xie , D. Liang , W. Shi , S. Chen , G. Li , W. Tang , C. Liu , Q. He , Nat. Commun. 2023, 14, 7783. 38012166 10.1038/s41467-023-43618-z PMC10682449

[21] J. Slezak , B. Kura , T. W. LeBaron , P. K. Singal , J. Buday , M. Barancik , Curr. Pharm. Des. 2021, 27, 610. 32954996 10.2174/1381612826666200821114016

[22] Y. Yu , Y. Yang , Y. Bian , Y. Li , L. Liu , H. Zhang , K. Xie , G. Wang , Y. Yu , Shock 2017, 48, 364. 28234792 10.1097/SHK.0000000000000856

[23] T. Kawamura , N. Wakabayashi , N. Shigemura , C.‐S. Huang , K. Masutani , Y. Tanaka , K. Noda , X. Peng , T. Takahashi , T. R. Billiar , M. Okumura , Y. Toyoda , T. W. Kensler , A. Nakao , Am. J. Pysiol. Lung Cell. Mol. Physiol. 2013, 304, L646. 10.1152/ajplung.00164.2012 PMC3652058 23475767

[24] Y. Zheng , Z. Zhang , T. Wang , J. Zhang , D. Tian , X. Zhang , Z. Wu , Chem. Eng. J. 2021, 425, 131800.

[25] Y. Ji , Y. Hu , Y. Feng , L. Liu , Z. Chen , H. Shen , Y. Han , H. Xu , L. Lao , Biomaterials 2025, 313, 122764. 39190941 10.1016/j.biomaterials.2024.122764

[26] D. Wu , M. Liang , H. Dang , F. Fang , F. Xu , C. Liu , Biochem. Biophy. Res. Commun. 2018, 495, 1620. 10.1016/j.bbrc.2017.11.193 29198702

[27] L. Li , X. Li , Z. Zhang , L. Liu , T. Liu , S. Li , S. Liu , Y. Zhou , F. Liu , Curr. Mol. Med. 2020, 20, 396. 31702499 10.2174/1566524019666191105150709

[28] W. Fang , G. Wang , L. Tang , H. Su , H. Chen , W. Liao , J. Xu , J. Cell Mol. Med. 2018, 22, 4243. 29921037 10.1111/jcmm.13704 PMC6111801

[29] A. Haraji , V. Rakhshan , V. Hosseini , Brit. J. Oral Max. Surg. 2016, 54, 266. 10.1016/j.bjoms.2016.01.020 26872898

[30] L. C. Kloth , J. E. Berman , C. H. Sutton , S. Dumit‐Minkel , P. E. Papanek , D. J. Wurzel , Symp. Therm. Regul. Wound Care. 2000, 237, 43. 11074989

[31] Y. Xu , M. Fan , W. Yang , Y. Xiao , L. Zeng , X. Wu , Q. Xu , C. Su , Q. He , Adv. Mater. 2021, 33, 2101455. 10.1002/adma.202101455 34369623

[32] X. Li , B. Zhou , M. Fan , C. Xia , F. Xu , Q. He , Nano Energy 2024, 131, 110235.

[33] B. Yuan , Z. Luo , W. Luan , L. Cao , R. Zhu , J. Mater. Res. 2023, 38, 3628.

[34] H. M. Sung‐Suh , J. R. Choi , H. J. Hah , S. M. Koo , Y. C. Bae , J. Photochem. Photobiol. A: Chem. 2004, 163, 37.

[35] K. Zhang , L. Wang , J. K. Kim , M. Ma , G. Veerappan , C.‐L. Lee , K.‐J. Kong , H. Lee , J. H. Park , Energy Environ. Sci. 2016, 9, 499.

[36] V. Schubert , Photodermatol. Photoimmunol. Photomed. 2001, 17, 32. 11169174 10.1034/j.1600-0781.2001.017001032.x

