# Supplementary material for: Local Visible‐Photocatalytic Production of Hydrogen and Warm Heat for Combination Treatment of Pressure Ulcer
Source: Adv Sci (Weinh). 2025 Jun 10;12(33):e03185. doi: 10.1002/advs.202503185 (PMC12412477; doi:10.1002/advs.202503185)
Supplement: Supplementary file 1 — Supporting Information [file ADVS-12-e03185-s001.pdf]

ADVANCED SCIENCE
Open Access

Supporting Information

for *Adv. Sci.*, DOI 10.1002/advs.202503185

Local Visible-Photocatalytic Production of Hydrogen and Warm Heat for Combination Treatment of Pressure Ulcer

Jiawei Zhu, Ting Chen, Wei Fang, Di Zhou, Dalong Ni, Bin Zhao, Yali Yang*, Zhuobin Xu* and Qianjun He**

Supplementary Information

Local Visible-Photocatalytic Production of Hydrogen and Warm Heat for Combination Treatment of Pressure Ulcer

Jiawei Zhu,[1,†] Ting Chen,[2,†] Wei Fang,[3,†] Di Zhou,[4,] Dalong Ni,[5] Bin Zhao,[2] Yali Yang,[6,*] Zhuobin Xu,[1,*] Qianjun He[7,8,*]*

[1] *Institute of Translational Medicine, School of Medicine, Yangzhou University, Yangzhou 225001, China*

[2] *Guangdong Key Laboratory for Biomedical Measurements and Ultrasound Imaging, National-Regional Key Technology Engineering Laboratory for Medical Ultrasound, School of Biomedical Engineering, Shenzhen University Medical School, Shenzhen 518060, China*

[3] *Department of Laser and Aesthetic Medicine, Shanghai Ninth People's Hospital, Shanghai JiaoTong University School of Medicine, Shanghai 200011, China*

[4] *Department of Radiology, The First Affiliated Hospital of Chongqing Medical University, Chongqing, China*

[5] *Department of Orthopaedics, Shanghai Key Laboratory for Prevention and Treatment of Bone and Joint Diseases, Shanghai Institute of Traumatology and Orthopaedics, Ruijin Hospital, Shanghai Jiao Tong University School of Medicine, Shanghai 200025, PR China*

[6] *Department of Dermatology, Shanghai Ninth People's Hospital, Shanghai Jiao Tong University School of Medicine, Shanghai 200011, China*

[7] *Shanghai Key Laboratory of Hydrogen Science & Center of Hydrogen Science, School of Materials Science and Engineering, Shanghai Jiao Tong University, Shanghai 200240, China*

[8] *Shenzhen Research Institute, Shanghai Jiao Tong University, Shenzhen 518057, China*

[†] These authors contributed equally to this work.

* Corresponding author. Qianjun He (qjhe@sjtu.edu.cn), Yali Yang (ky7812@126.com), and Zhuobin Xu (xuzb@yzu.edu.cn)

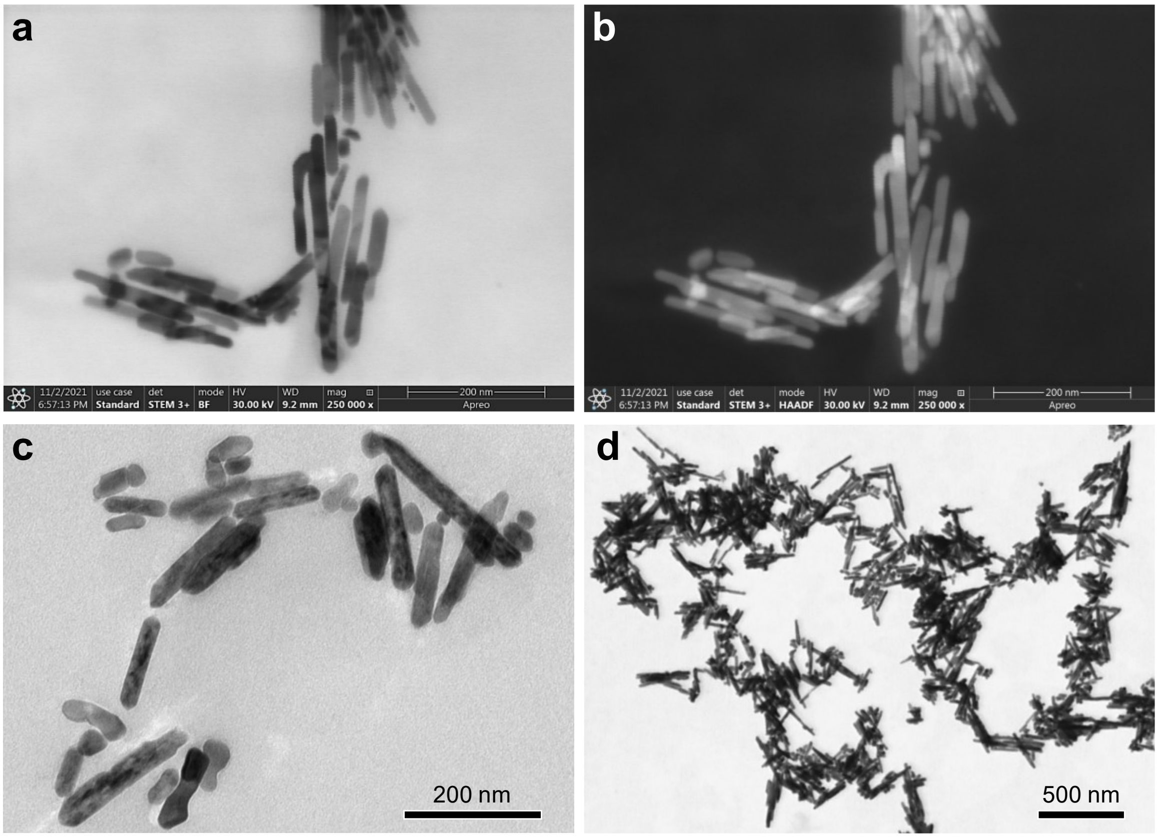

Figure S1. TEM images of TON (**a**,**b**) and HTON (**c**,**d**).

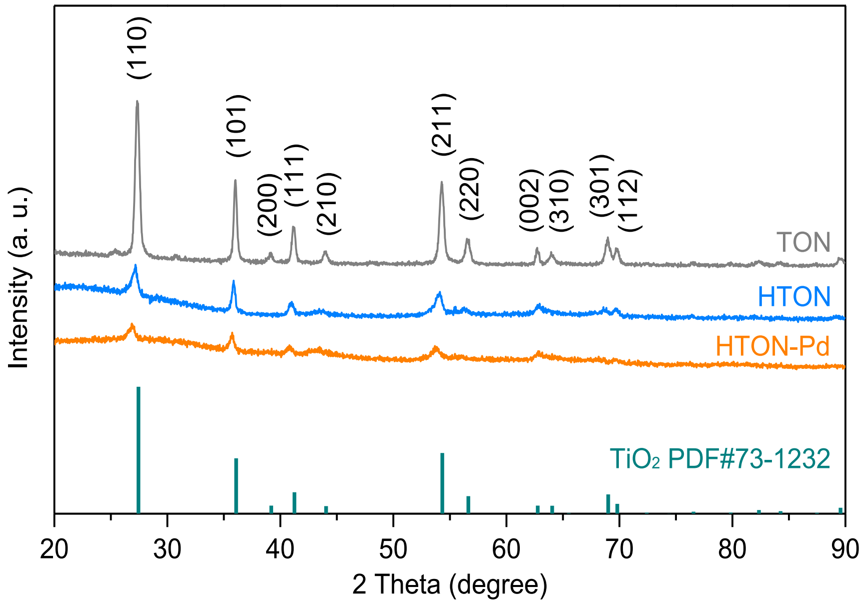

Figure S2. The XRD patterns of TON, HTON, and HTON-Pd powders.

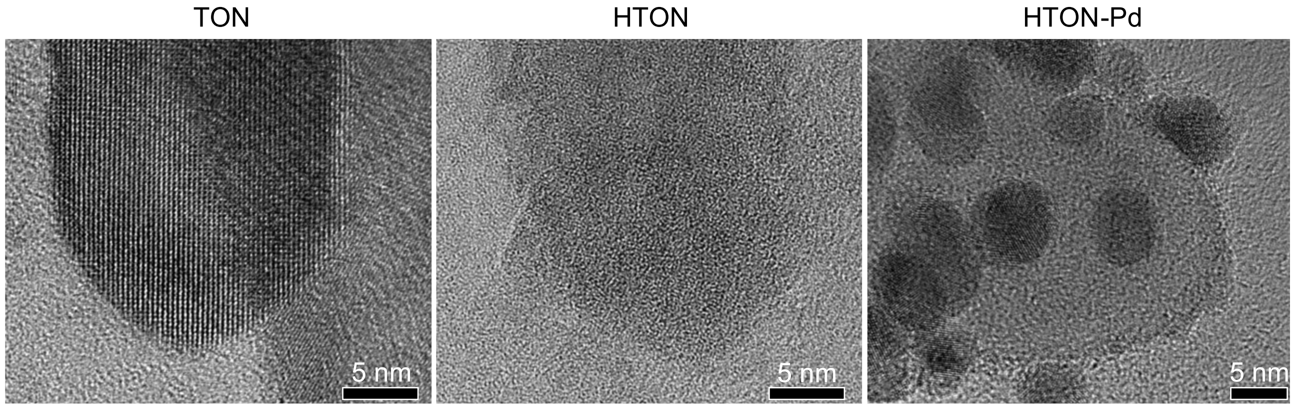

Figure S3. The high-resolution TEM images of TON, HTON, and HTON-Pd.

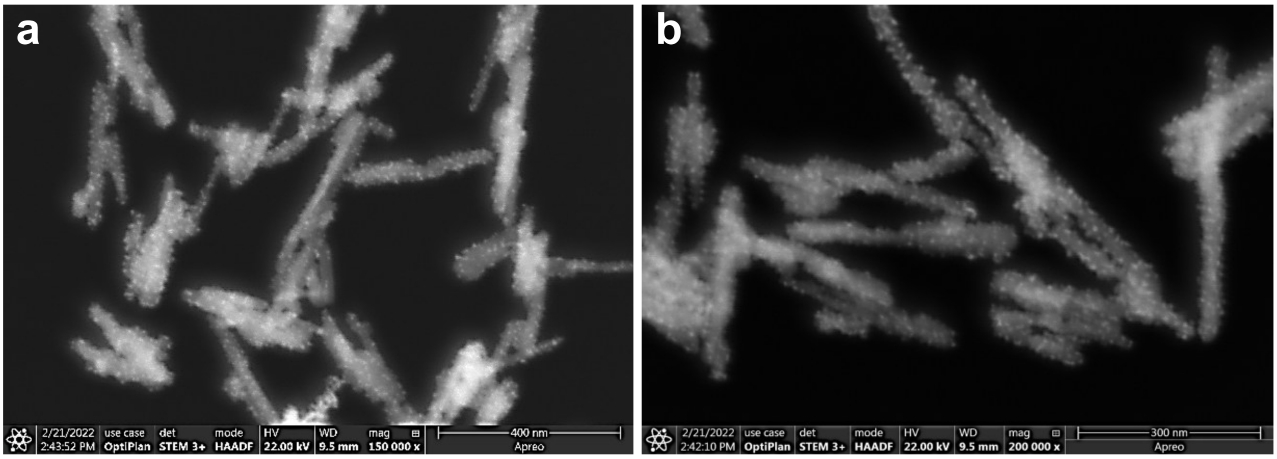

Figure S4. HADDF images of HTON-Pd.

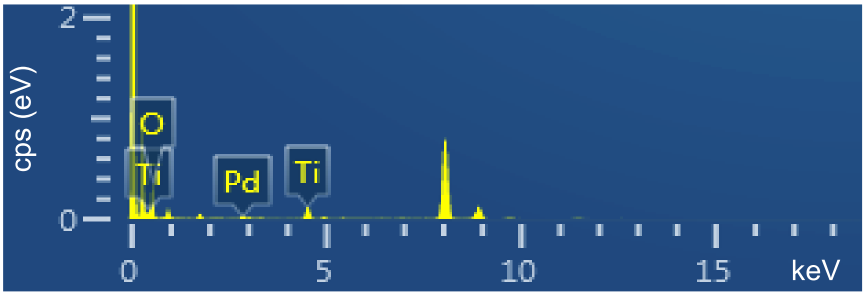

Figure S5. The EDS pattern of HTON-Pd.

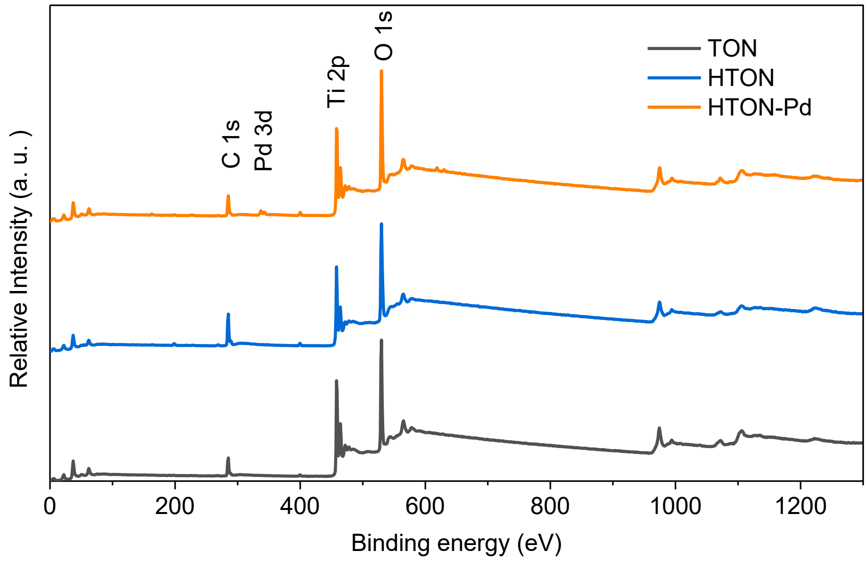

Figure S6. XPS patterns of TON, HTON, and HTON-Pd.

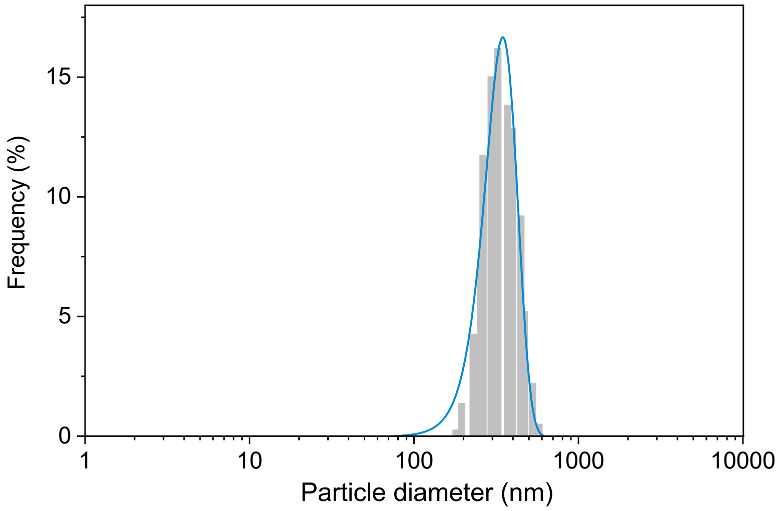

Figure S7. The DLS result of HTON-Pd.

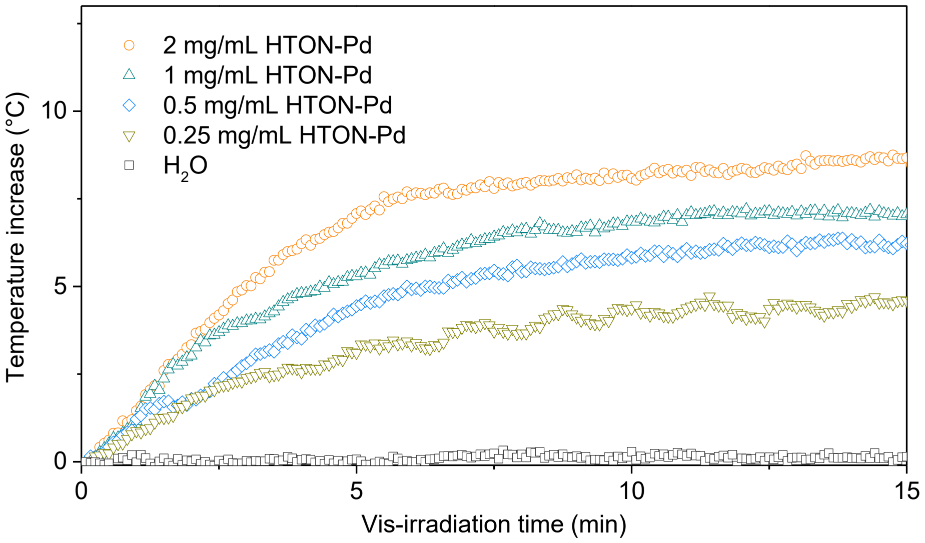

Figure S8. Photothermal curves of HTON-Pd at different concentrations under irradiation with a xenon lamp at a power density of 0.05 W/cm^2.

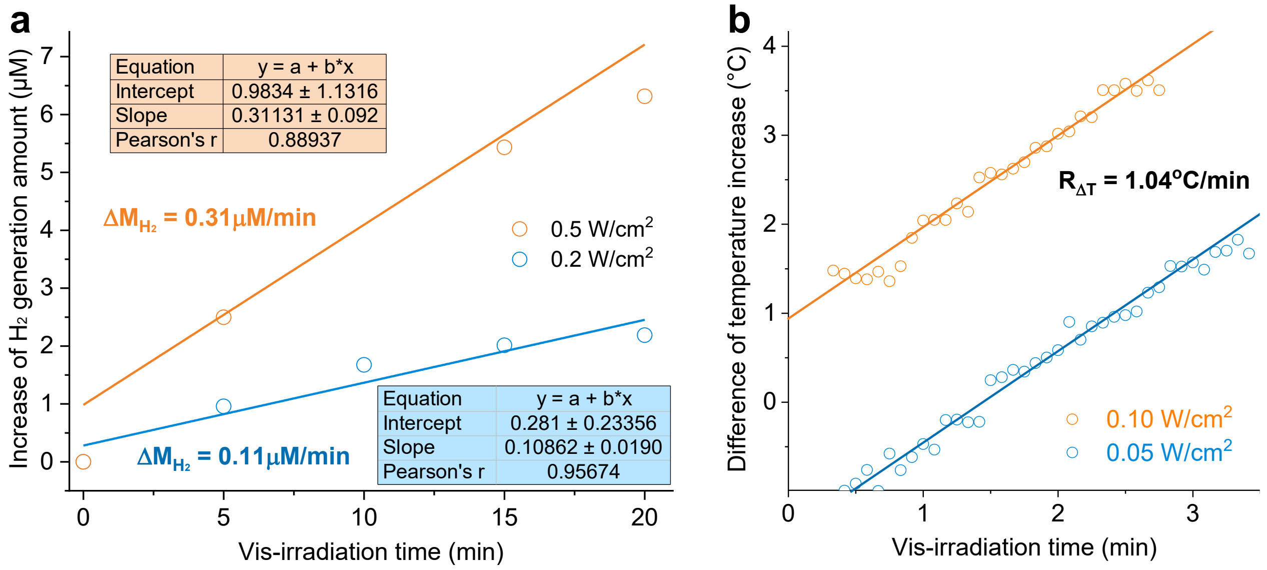

Figure S9. The efficiency and stability of HTON-Pd for visible-photocatalytic generation of H_2 (**a**) and heat (**b**) at different power densities of visible light.

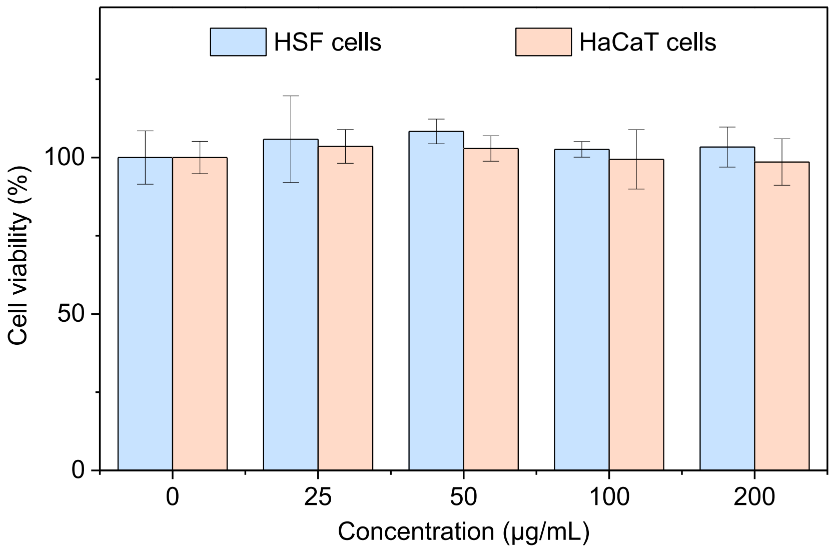

Figure S10. The cytotoxicity of HTON-Pd nanorods to HSF and HaCaT cells (*n* = 6, biologically independent samples).

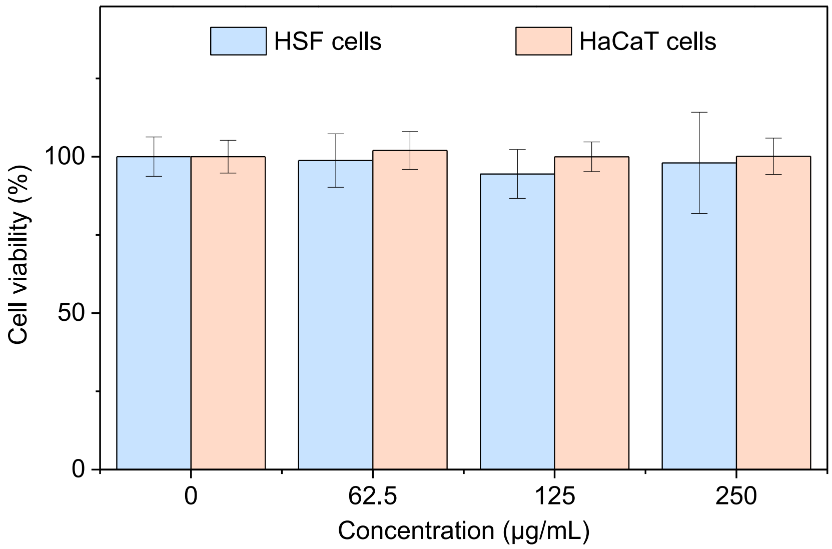

Figure S11. The cytotoxicity of GSH to HSF and HaCaT cells (*n* = 6, biologically independent samples).

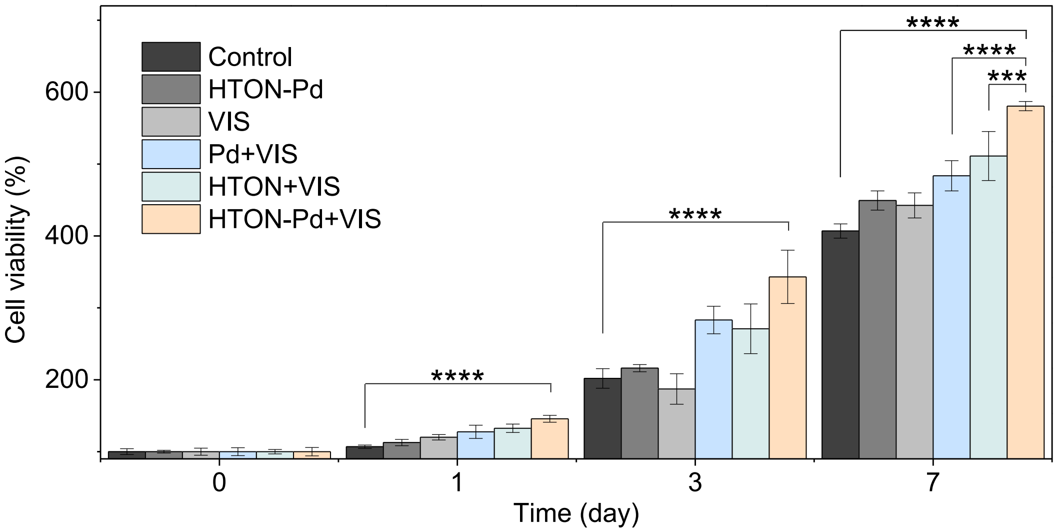

Figure S12. The effect of hydrogen and warm heat for combination treatment on the proliferation of HaCaT cells (n = 6, biologically independent samples).

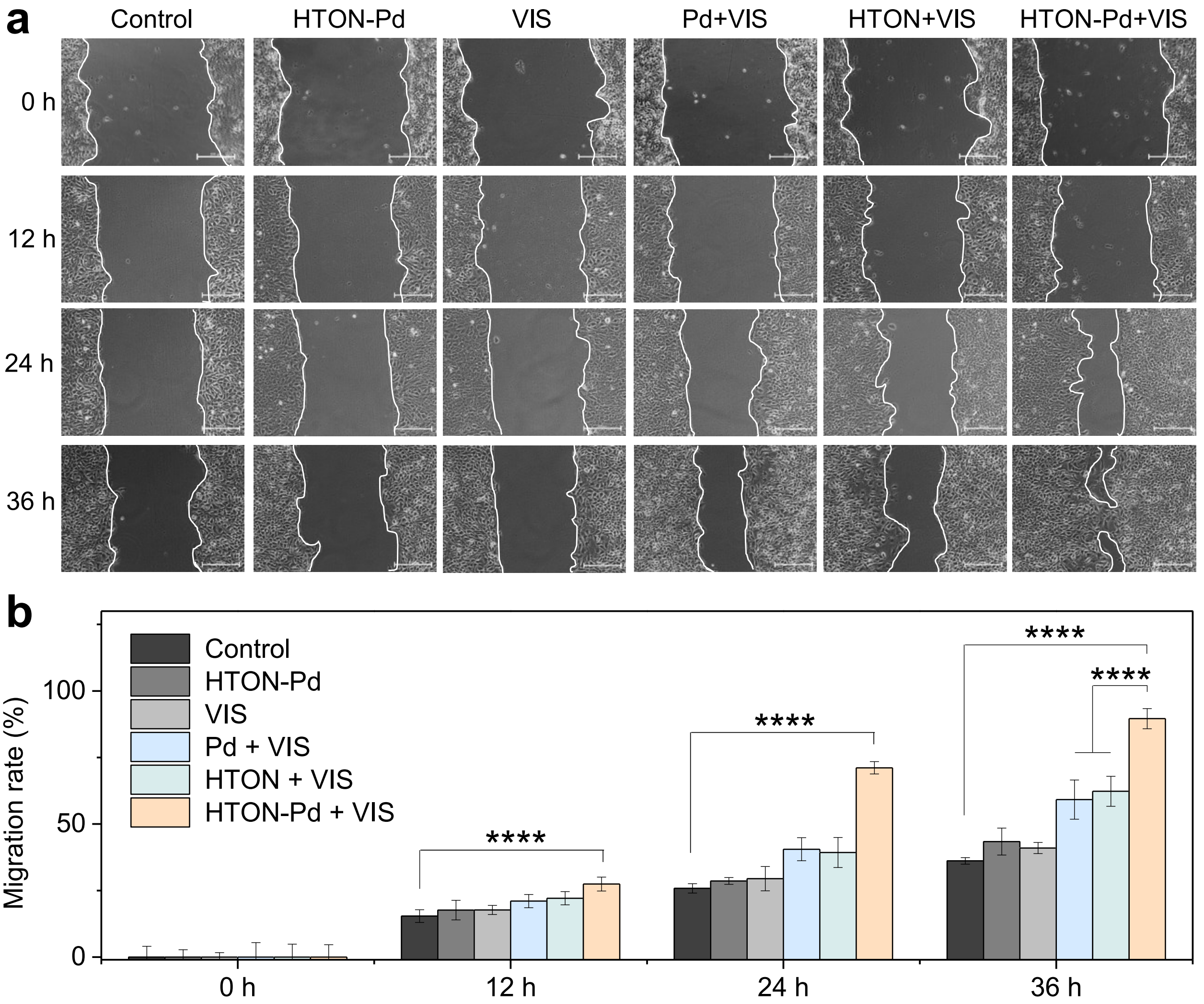

Figure S13. The effect of hydrogen and warm heat for combination treatment on the migration of HaCaT cells (**a**), and corresponding statistical analysis (n = 5, biologically independent samples) (**b**). Scale bar, 200 μm.

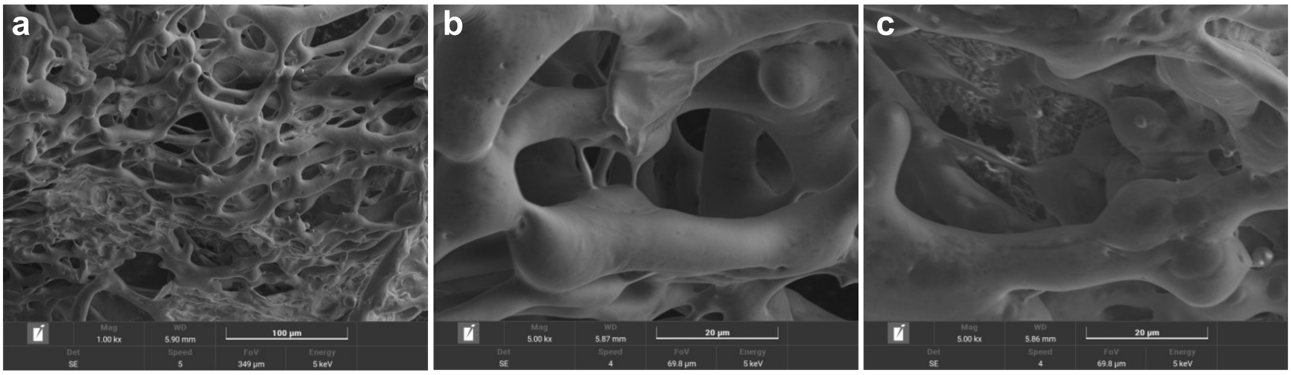

Figure S14. The SEM images of CS/HA hydrogel (**a**), and HTON-Pd@Gel before (**b**) and after 15-min light irradiation (**c**).

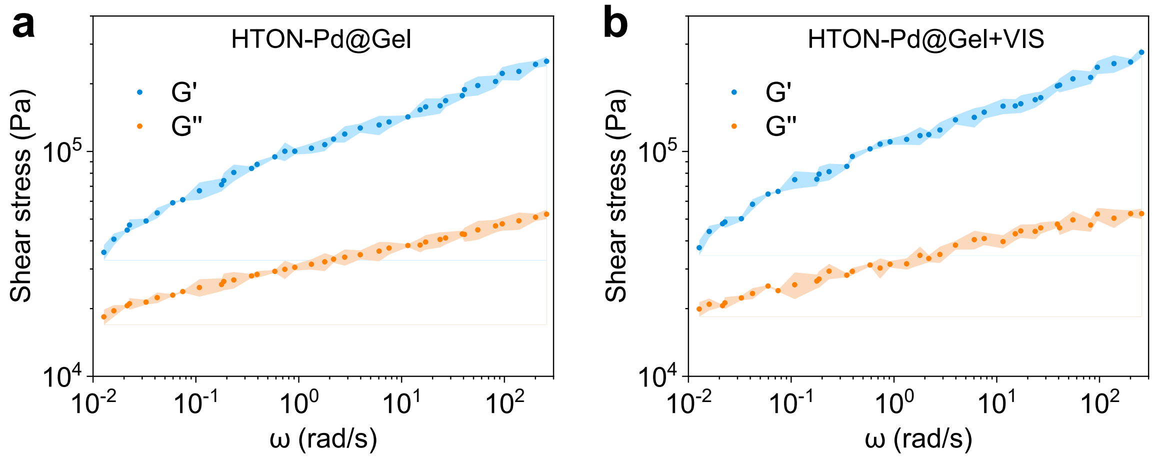

Figure S15. Comparison of rheological behaviors of HTON-Pd@Gel and HTON-Pd@Gel+VIS (n=3).

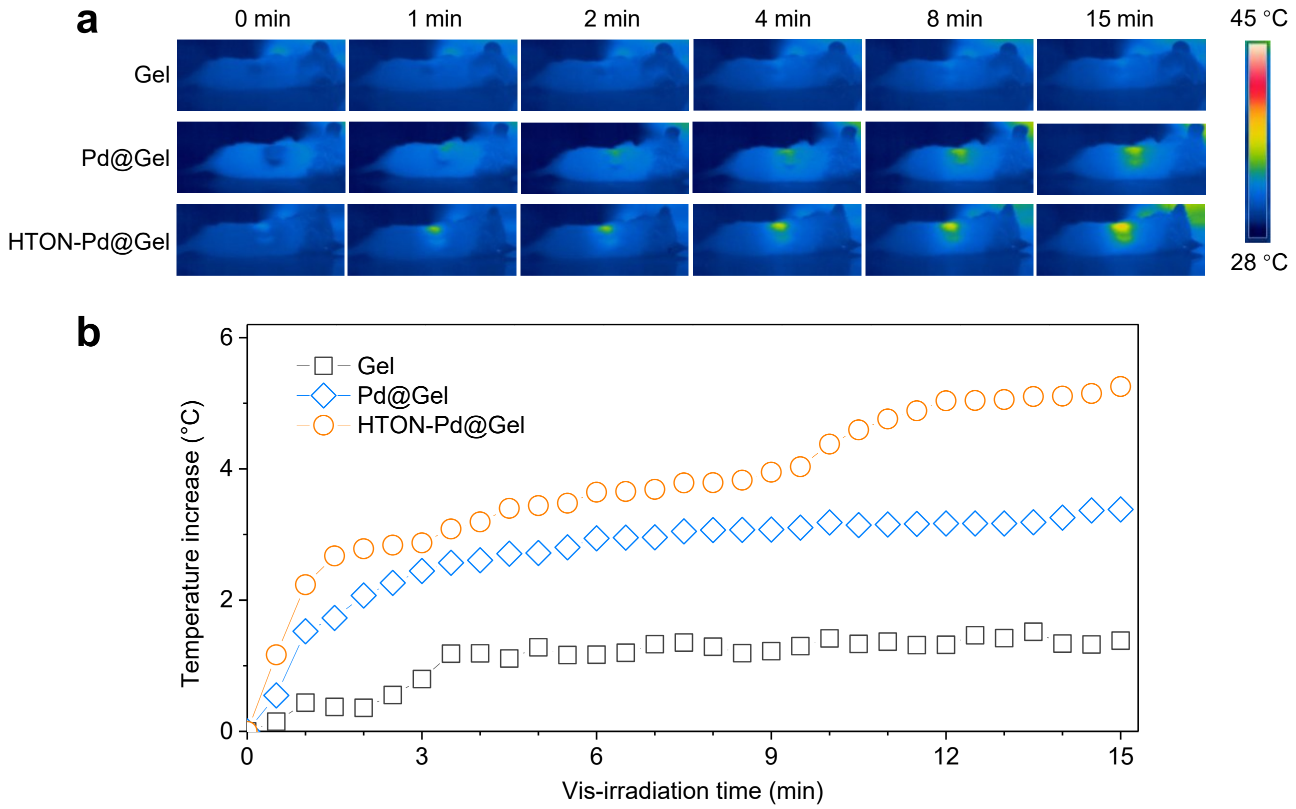

Figure S16. *In vivo* thermal imaging tracking of pressure ulcer mice with various treatments (**a**), and the temperature change at the pressure sore site (**b**).

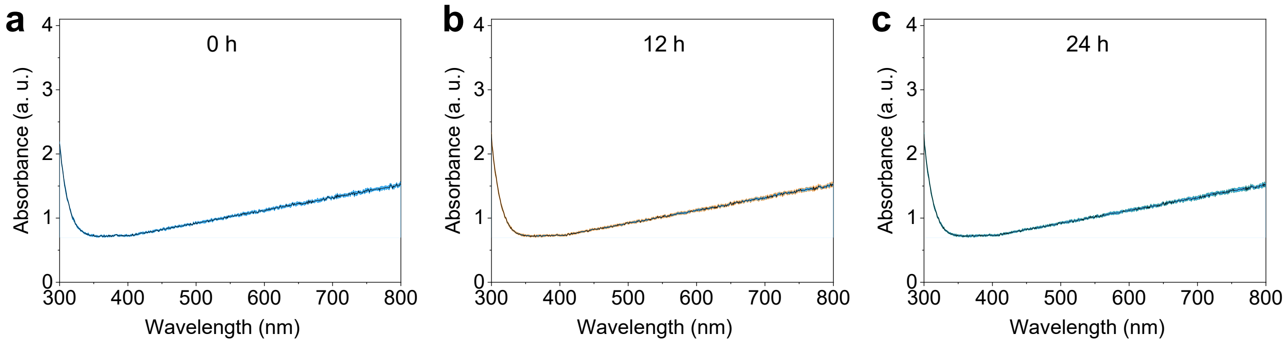

Figure S17. The UV-Vis absorption spectra of HTON-Pd@Gel after light illumination for 0 h (**a**), 12 h (**b**), and 24 h (**c**).

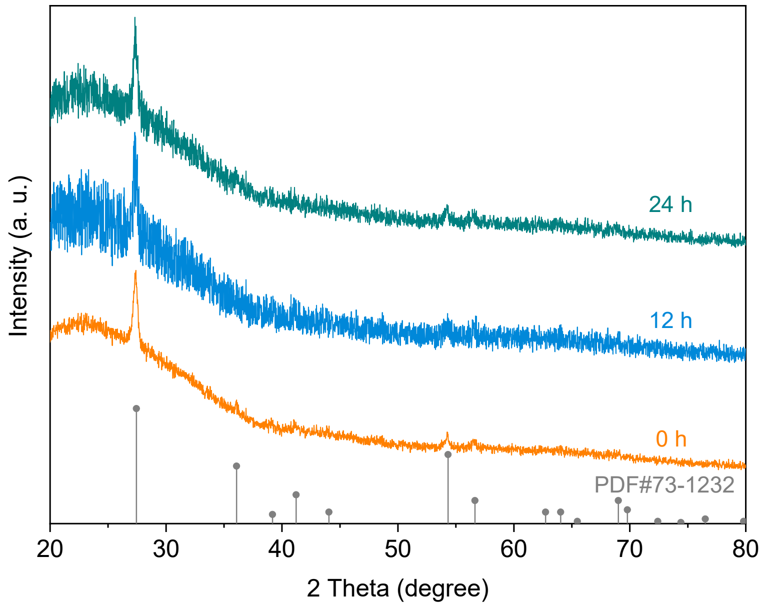

Figure S18. The XRD patterns of HTON-Pd@Gel after light illumination for 0 h, 12 h and 24 h.

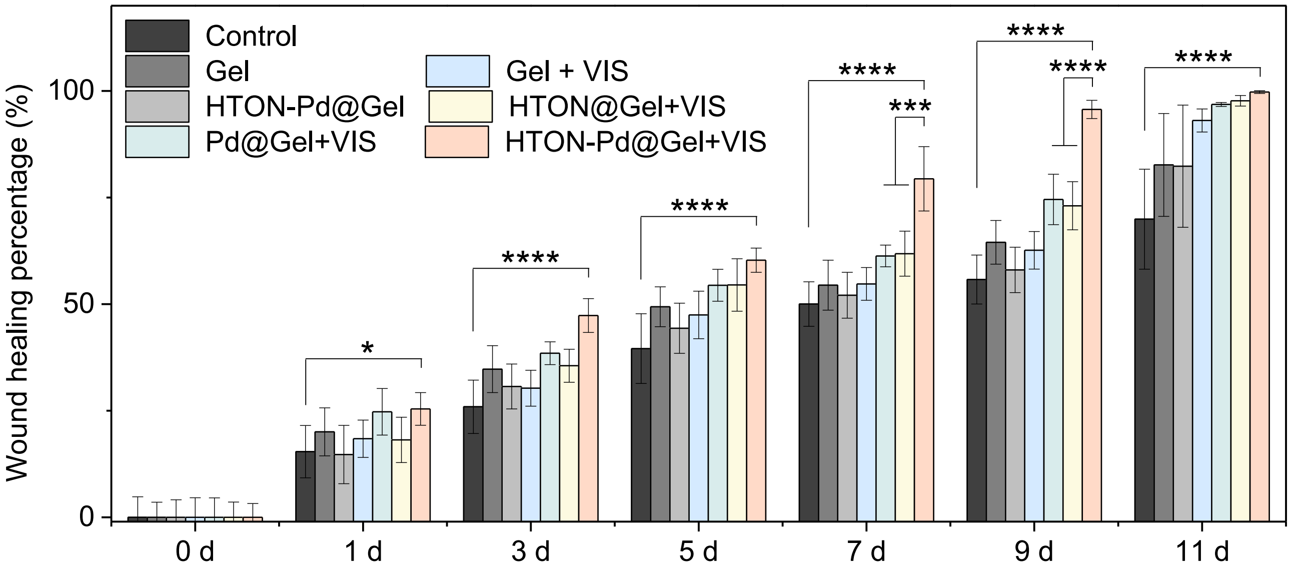

Figure S19. Statistical analysis of wound healing rate in mice (*n* = 6, biologically independent samples).

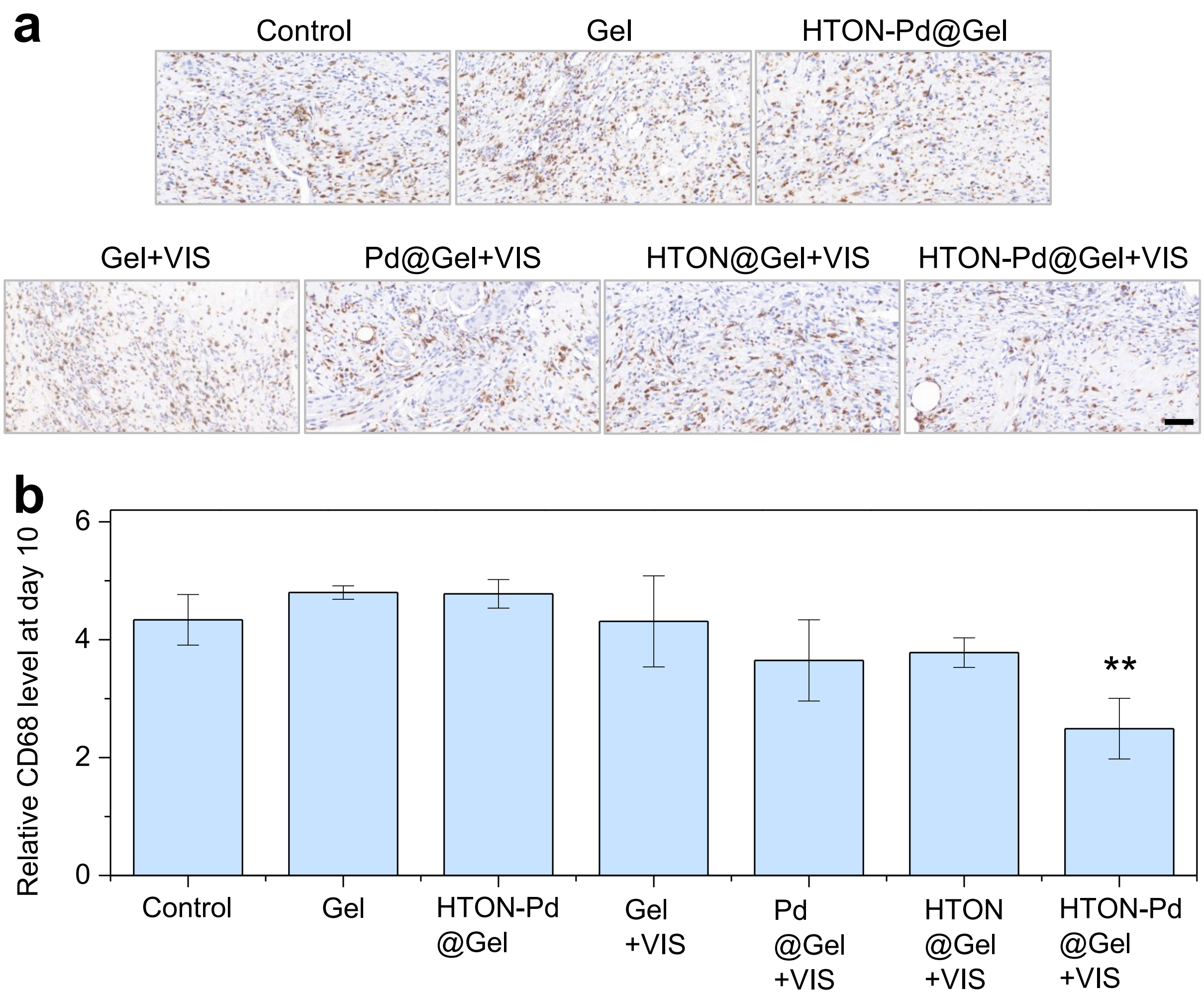

Figure S20. Immunohistochemical staining of CD68 expression on day 10 after treatment (**a**), and corresponding statistical analysis (n = 3 biologically independent samples) (**b**). Scale bar, 50 μm).

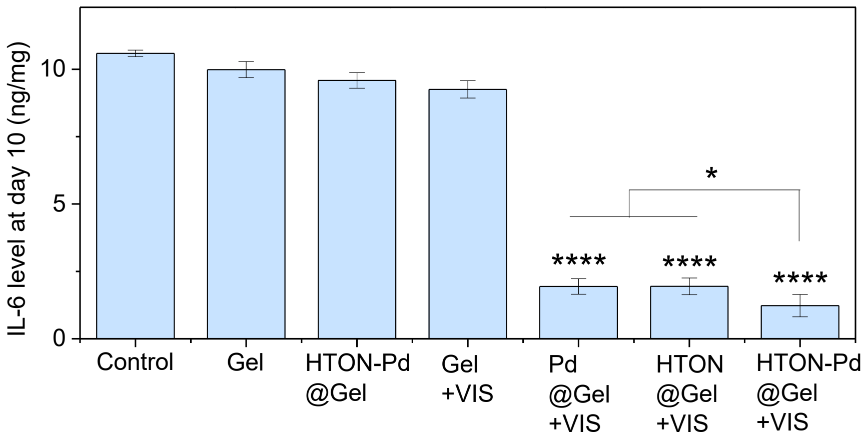

Figure S21. ELISA analysis of IL-6 expression at the wound site on day 10 after treatment (n = 4 biologically independent samples).

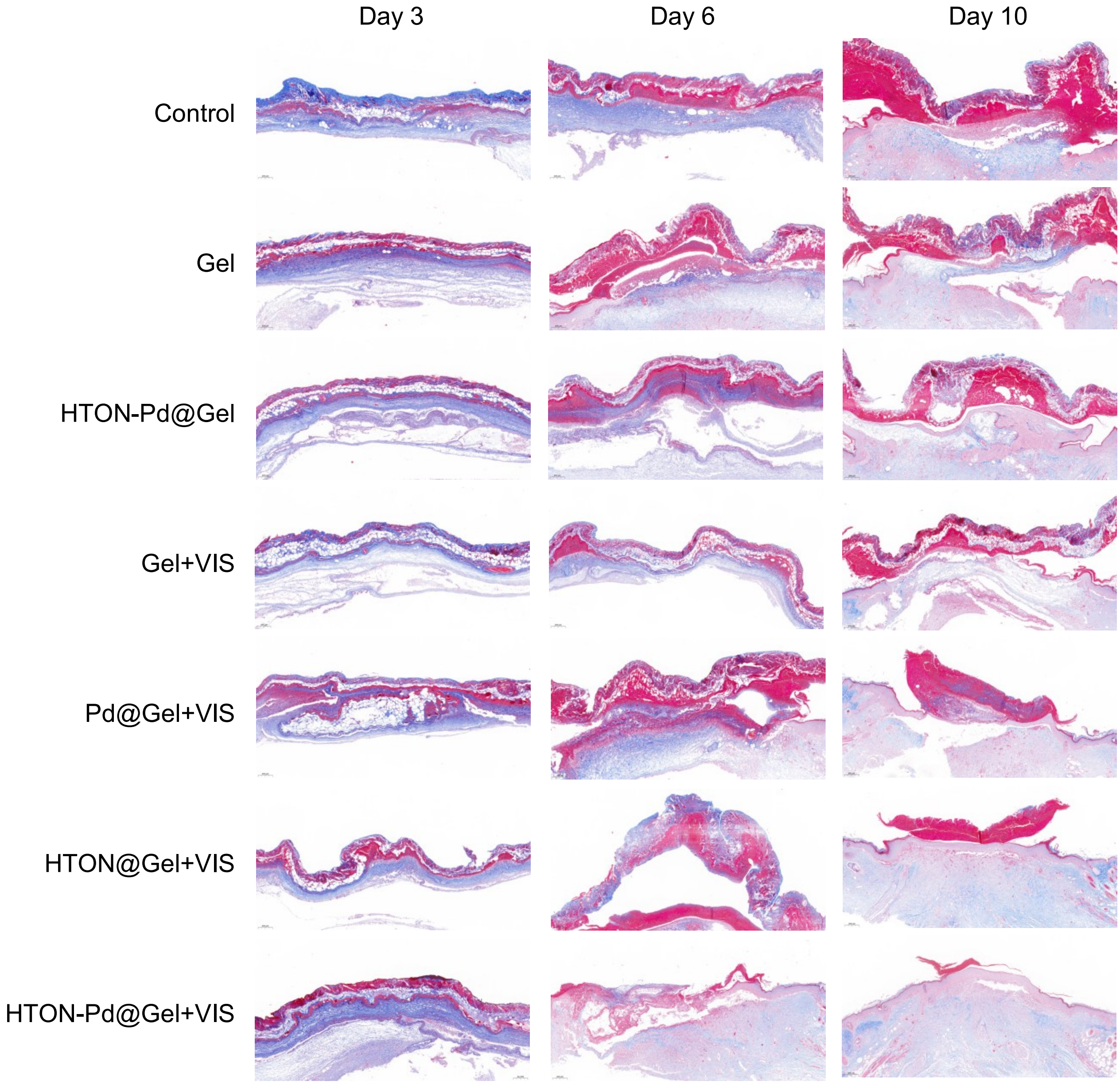

Figure S22. Masson images of wound tissues with various treatments.

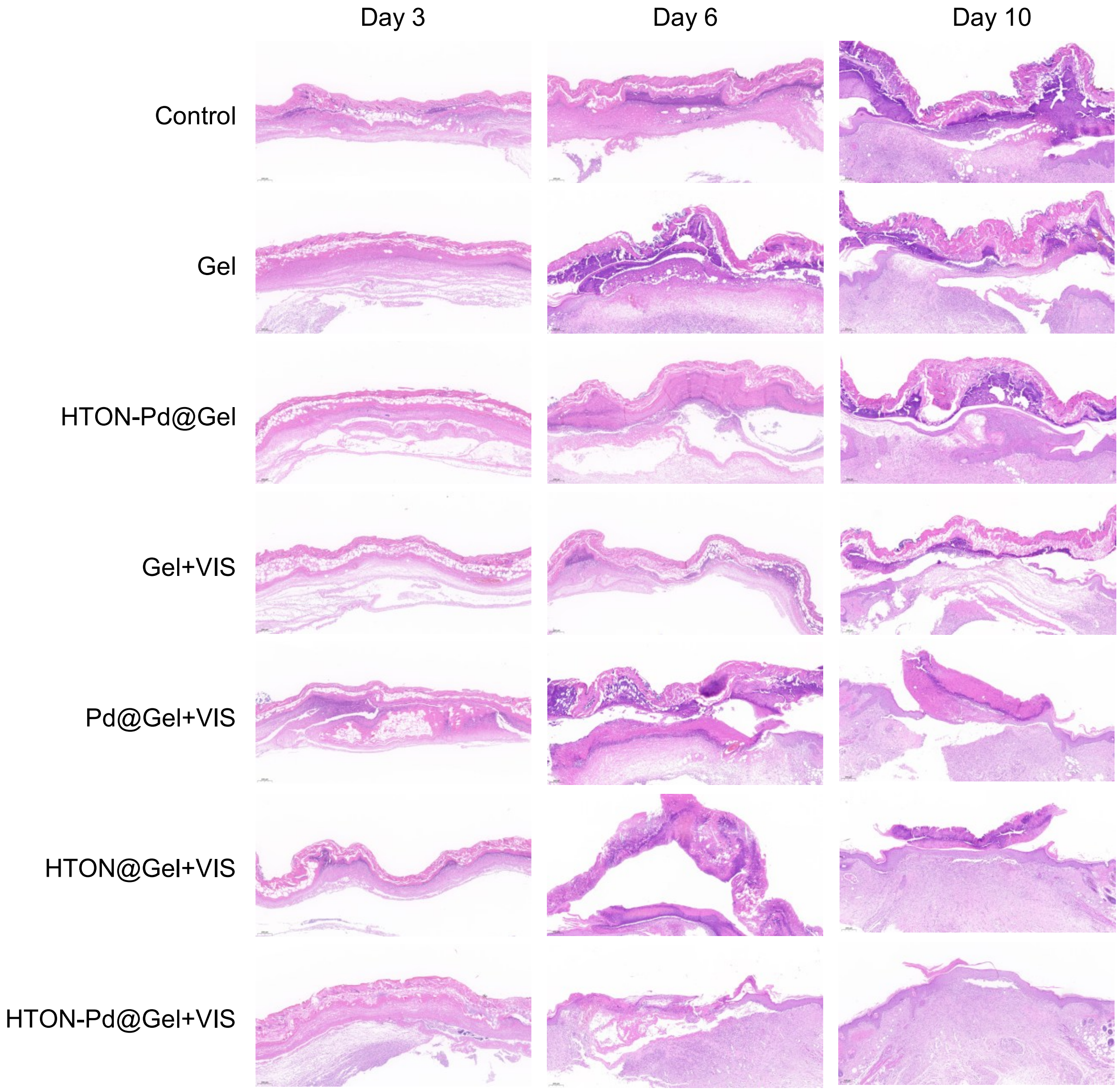

Figure S23. HE images of wound tissues with various treatments.

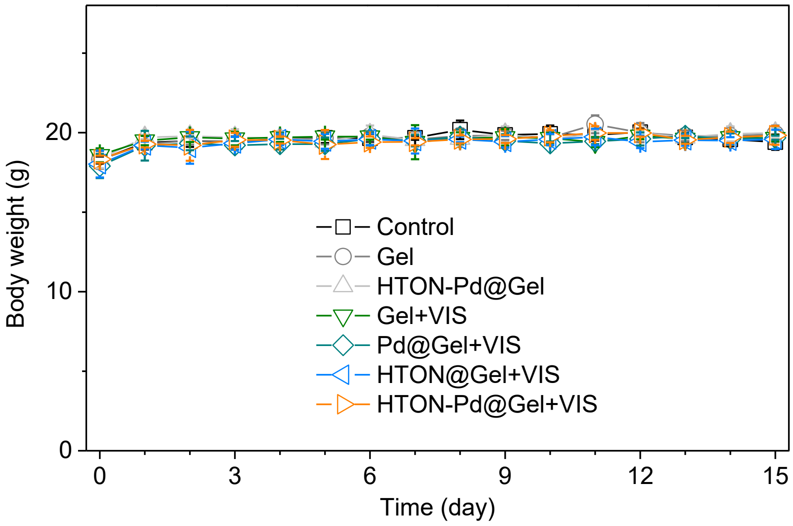

Figure S24. The change in the body weight of pressure ulcer mice during treatment (n = 4, biologically independent samples).

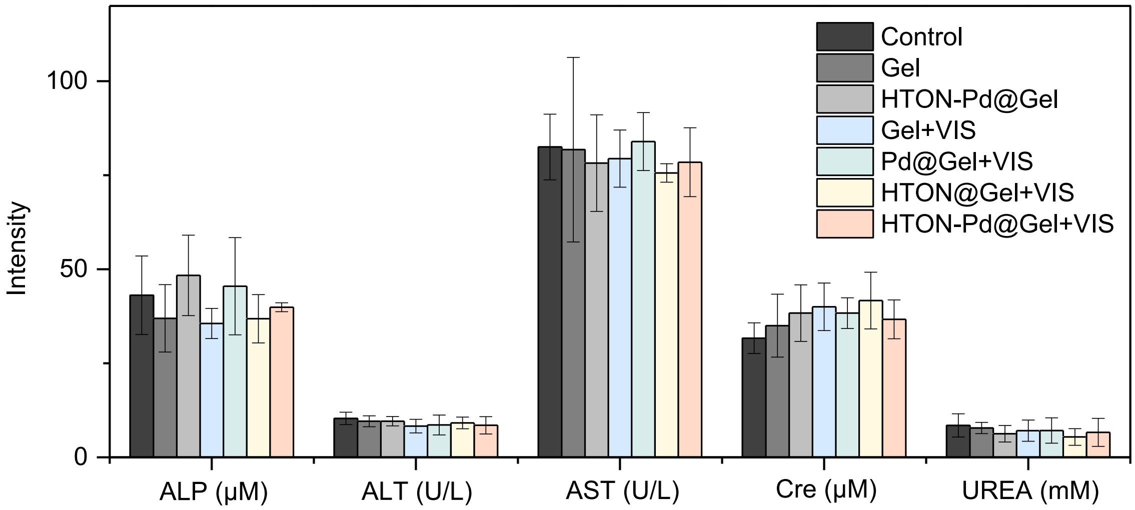

Figure S25. Blood biochemical analysis of liver and kidney functions in mice. ALP: alkaline phosphatase; ALT: Alanine transaminase; AST: aspartate aminotransferase; Cre: creatinine; UREA: urea nitrogen (n = 6, biologically independent samples).

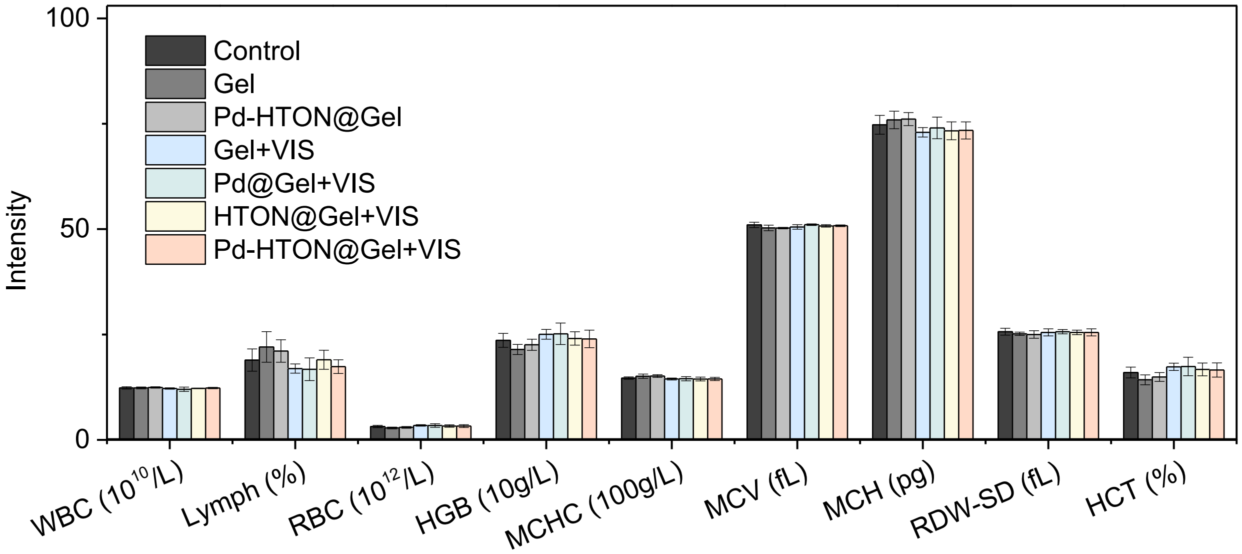

Figure S26. Analysis of blood routine biochemical indicators. WBC: leukocyte; Lymph: Lymphocyte percentage; RBC: red blood cell; HGB: hemoglobin concentration; MCHC: mean corpuscular hemoglobin concentration; MCV: mean red blood cell volume; MCH: mean corpuscular hemoglobin; RDW-SD: Red blood cell volume distribution width; HCT: hematocrit (n = 6, biologically independent samples).

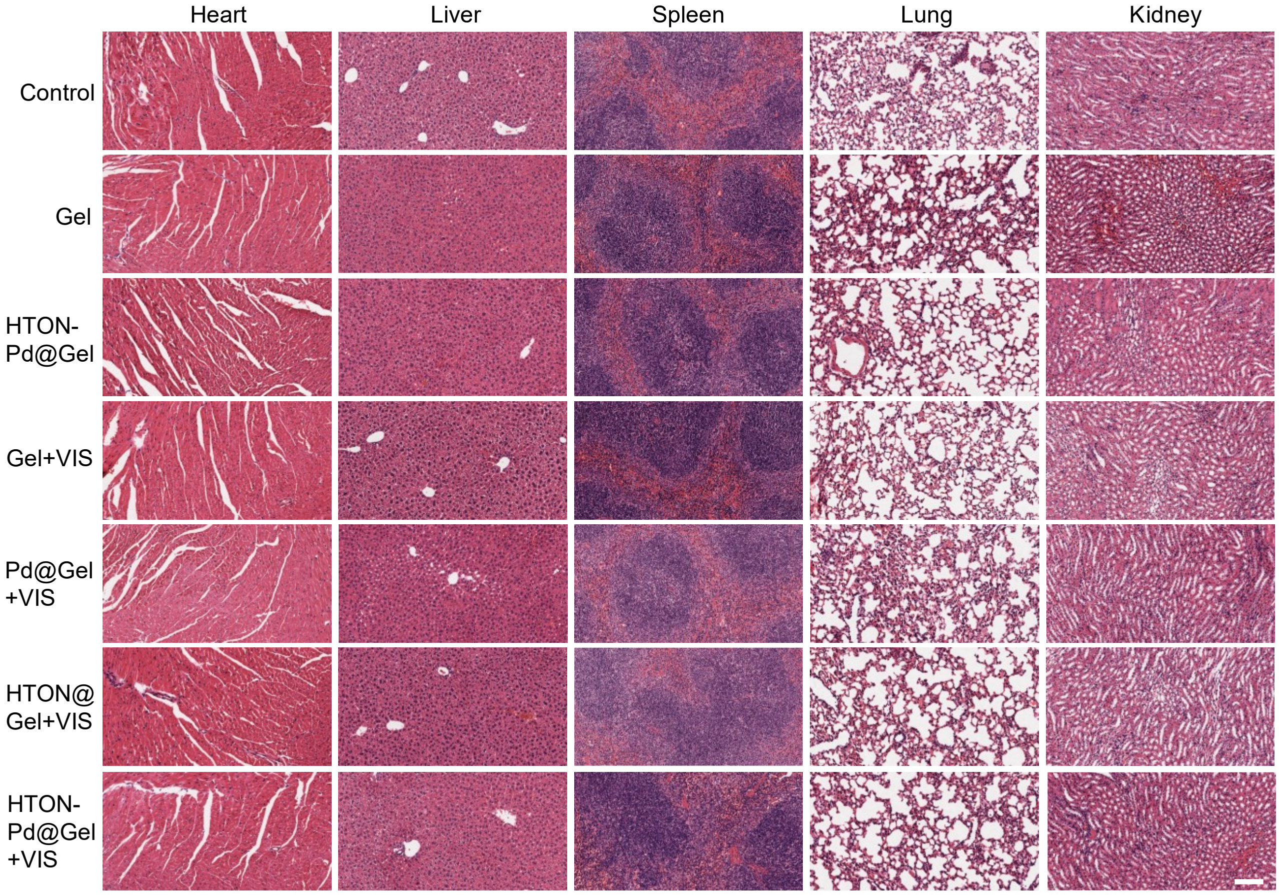

Figure S27. HE images of main organs (heart, lungs, liver, kidney, and spleen) at the end of treatment. Scale bar, 100 μm.

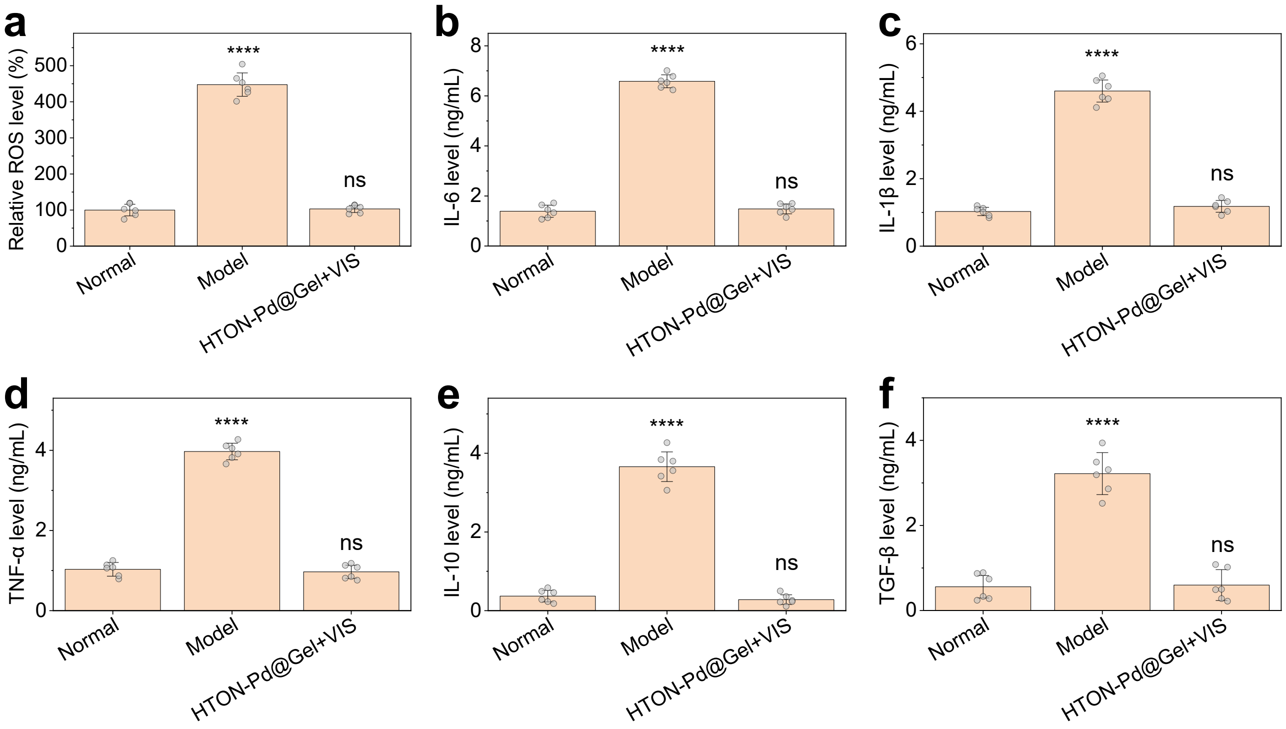

Figure S28. The levels of ROS (**a**), IL-6 (**b**), IL-1β (**c**), TNF-α (**d**), IL-10 (**e**), and TGF-β (**f**) at the wound site after treatment with HTON-Pd@Gel or HTON-Pd@Gel+VIS for two weeks (n = 6, biologically independent samples).

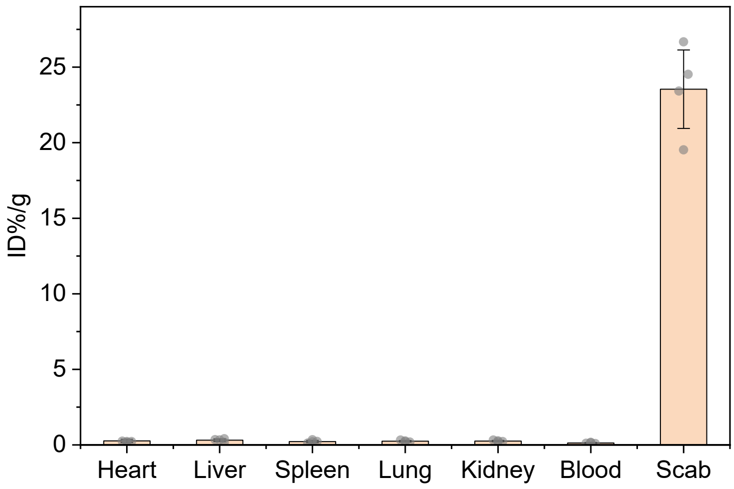

Figure S29. The biodistribution of palladium in pressure ulcer mice after HTON-Pd@Gel+VIS treatment for two weeks by ICP measurement (n = 4, biologically independent samples).
